# Supplementary material for: Novel Approach to the Construction of Fused Indolizine Scaffolds: Synthesis of Rosettacin and the Aromathecin Family of Compounds
Source: Molecules. 2023 May 12;28(10):4059. doi: 10.3390/molecules28104059 (PMC10222911; doi:10.3390/molecules28104059)

The novel approach to construction of fused indolizine scaffold: the synthesis of aromathecin family, rosettacin and its analogs

Shohta Mizuno, Takashi Nishiyama, Mai Endo, Koharu Sakoguchi, Takaki Yoshiura, Toshiyuki Motoyashiki, Noriyuki Hatae, Tominari Choshi

[1] Faculty of Pharmacy and Pharmaceutical Sciences, Fukuyama University, Fukuyama, Hiroshima 729-0292, Japan.
[2] Faculty of Pharmaceutical Sciences, Yokohama University of Pharmacy, 601 Matano, Totsukaku, Yokohama 245-0066, Japan.
Corresponding author: E-mail: choshi@fukuyama-u.ac.jp

Supporting Information

Copies of ^{1}H NMR and ^{13}C NMR Spectra of Synthesized Compounds

- 2-Iodo-3-methylquinoline (**20a**)
- 2-Iodoquinoline-3-carbaldehyde (**20b)**
- Methyl 2-iodoquinoline-3-carboxylate (**21**)
- 2-Iodoquinolin-3-ylmethanol (**22**)
- 2-Iodo-3-methoxymethylquinoline (**23**)
- Methyl 2-(2-formylphenyl)ethynylquinoline-3-carboxylate (**24a**)
- 2-(3-Methylquinolin-2-yl)ethynylbenzaldehyde (**24b**)
- 2-(3-Methoxymethylquinolin-2-yl)ethynylbenzaldehyde (**24c**)
- Methyl 2-(2-hydroxyiminomethylphenyl)ethynylquinoline-3-carboxylate (**25a**)
- 2-(3-Methylquinolin-2-yl)ethynylbenzaldehyde oxime (**25b**)
- 2-(3-Methoxymethylquinolin-2-yl)ethynylbenzaldehyde oxime (**25c**)
- 3-(3-Methylquinolin-2-yl)isoquinolin-1-one (**27b**)
- 4-Acetoxy-3-(3-methylquinolin-2-yl)isoquinoline (**28b**)
- 3-(3-Methoxymethylquinolin-2-yl)isoquinoline *N*-oxide (**26c**)
- 3-(3-Methoxymethylquinolin-2-yl)isoquinolin-1-one (**27c**)
- 4-Acetoxy-3-(3-methoxymethylquinolin-2-yl)isoquinoline (**28c**)
- 4-Hydroxy-3-(3-methoxymethylquinolin-2-yl)isoquinoline (**29**)
- Rosettacin (**5**)
- 3-Methoxymethyloxymethyl-2-[(3-methoxymethylquinolin-2-yl)ethynyl]benzaldehyde (**31**)
- 3-Methoxymethyloxymethyl-2-[(3-methoxymethylquinolin-2-yl)ethynyl]benzaldehyde oxime (**32**)
- 5-Methoxymethyloxymethyl-3-(3-methoxymethylquinolin-2-yl)isoquinolin-1-one (**34**)
- 4-Acetoxy-5-methoxymethyloxymethyl-3-(3-methoxymethylquinolin-2-yl)isoquinoline (**35**)
- Acuminatine (**7**)

2-Iodo-3-methylquinoline (20a)

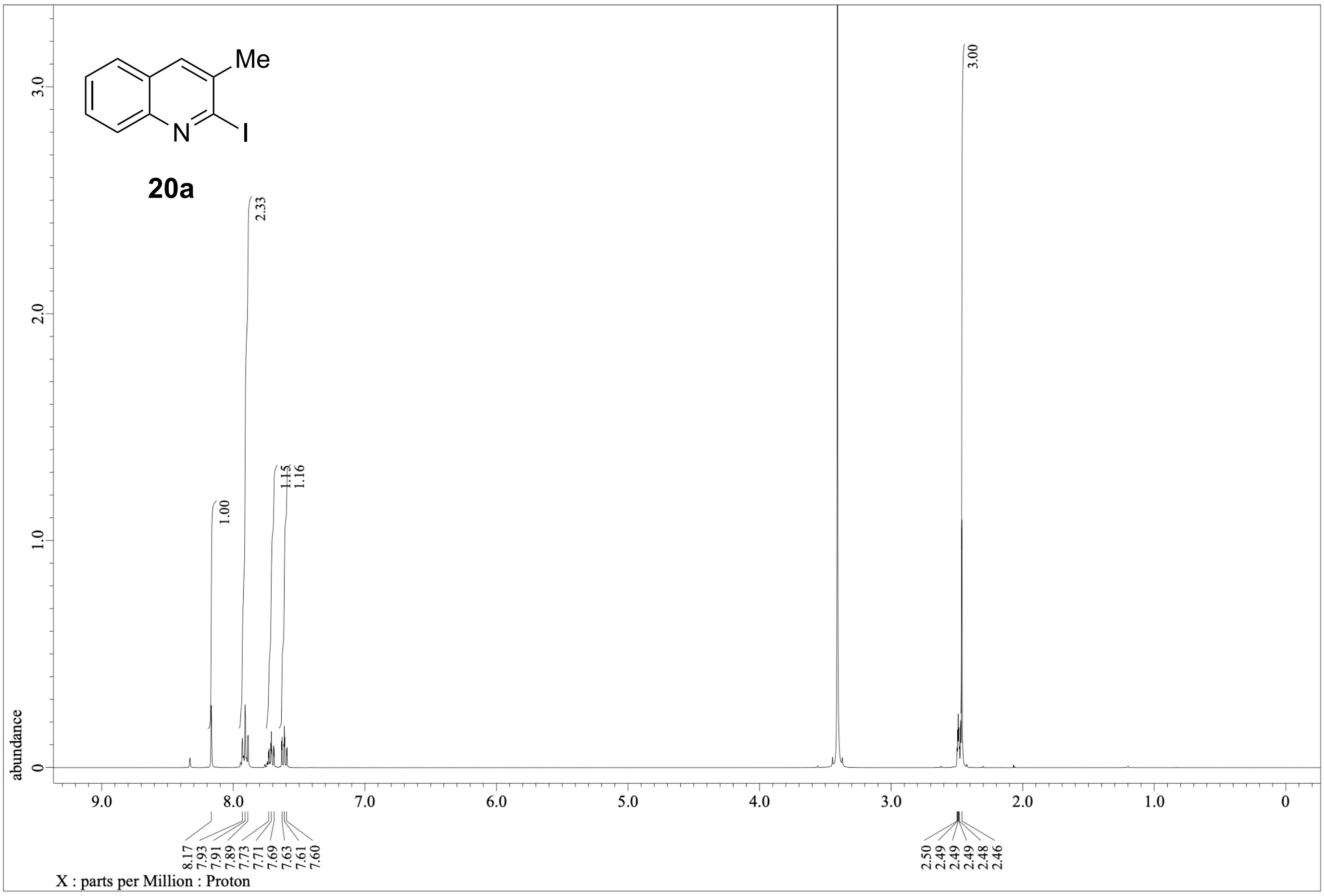

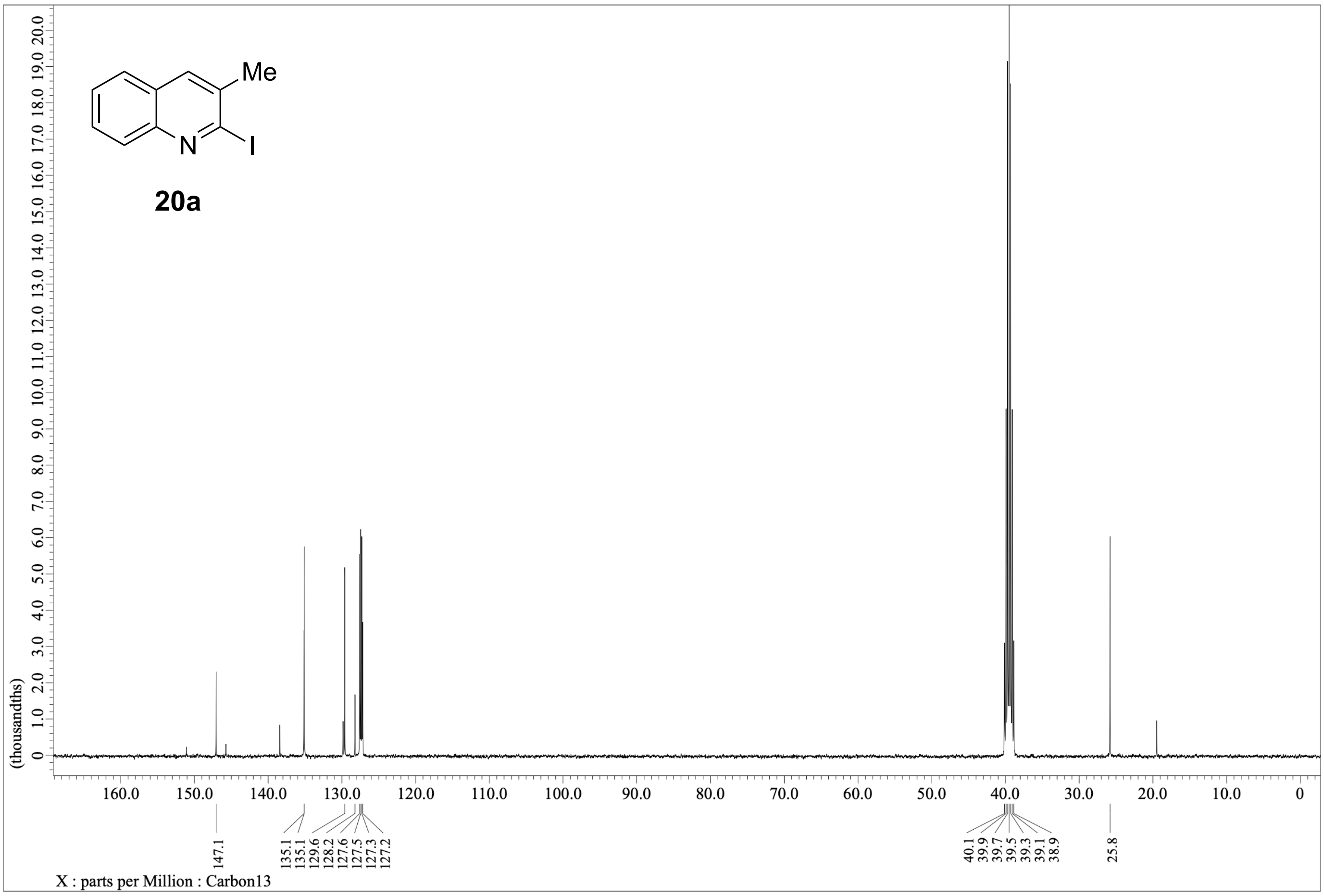

2-Iodoquinoline-3-carbaldehyde (20b)

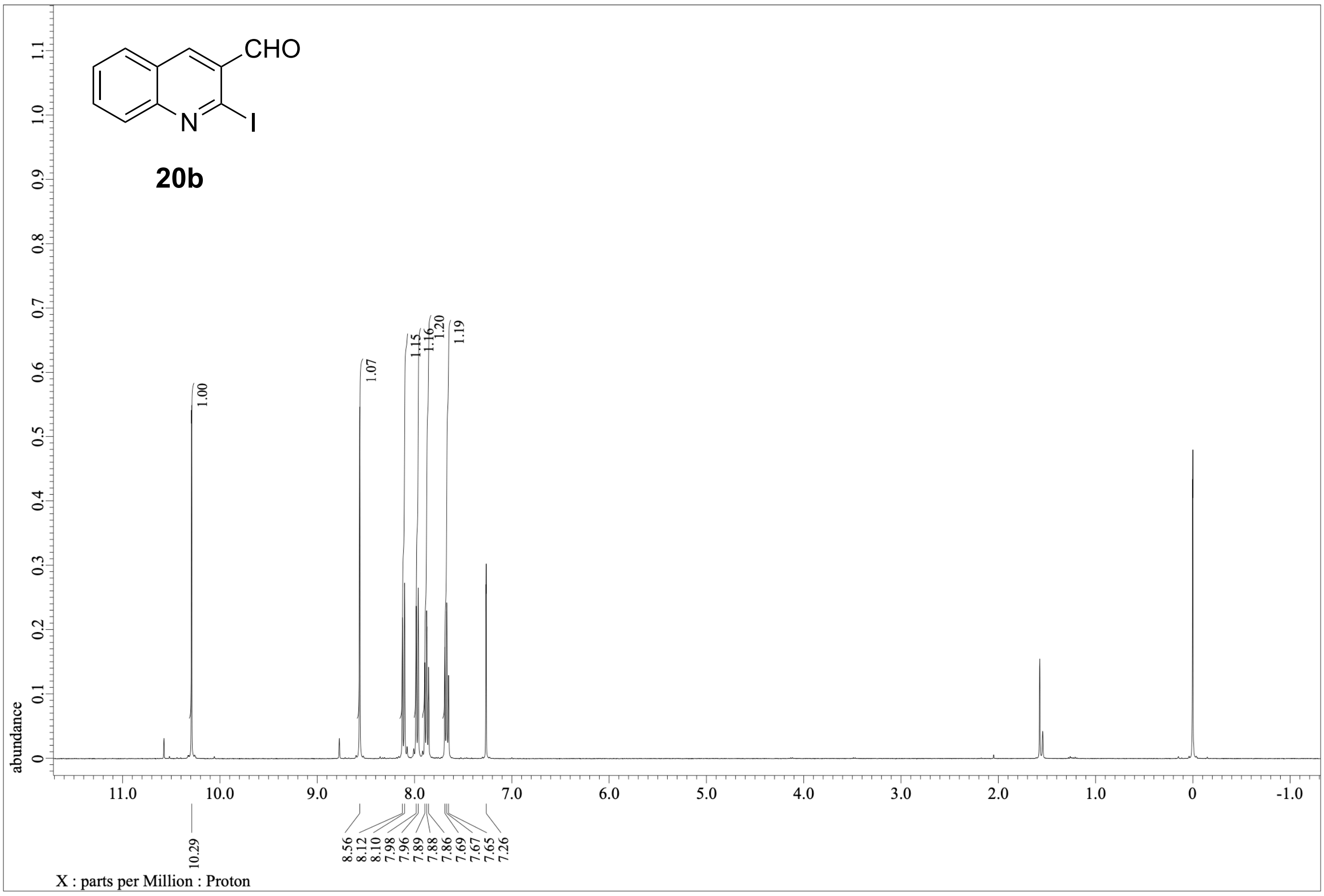

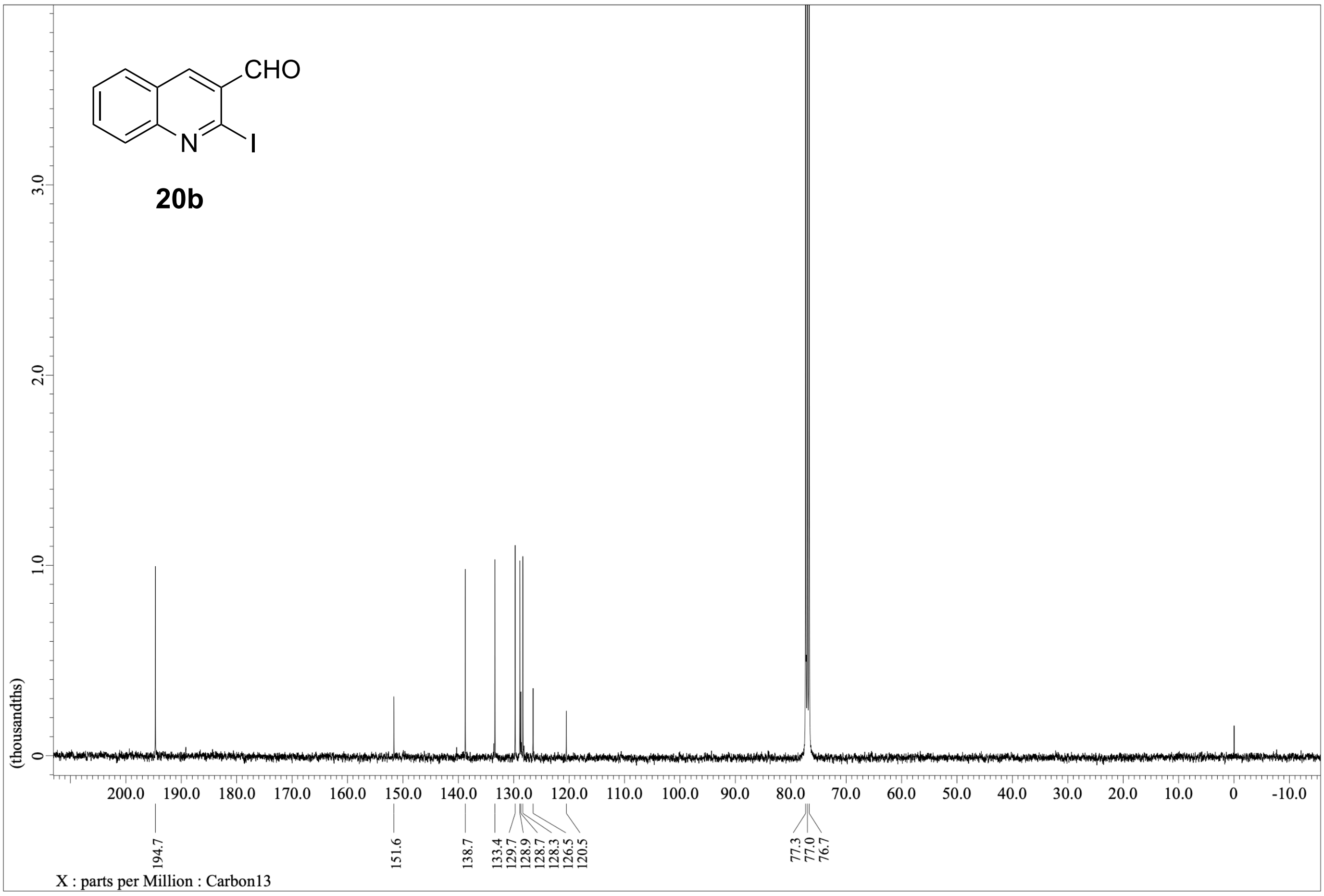

Methyl 2-iodoquinoline-3-carboxylate (21)

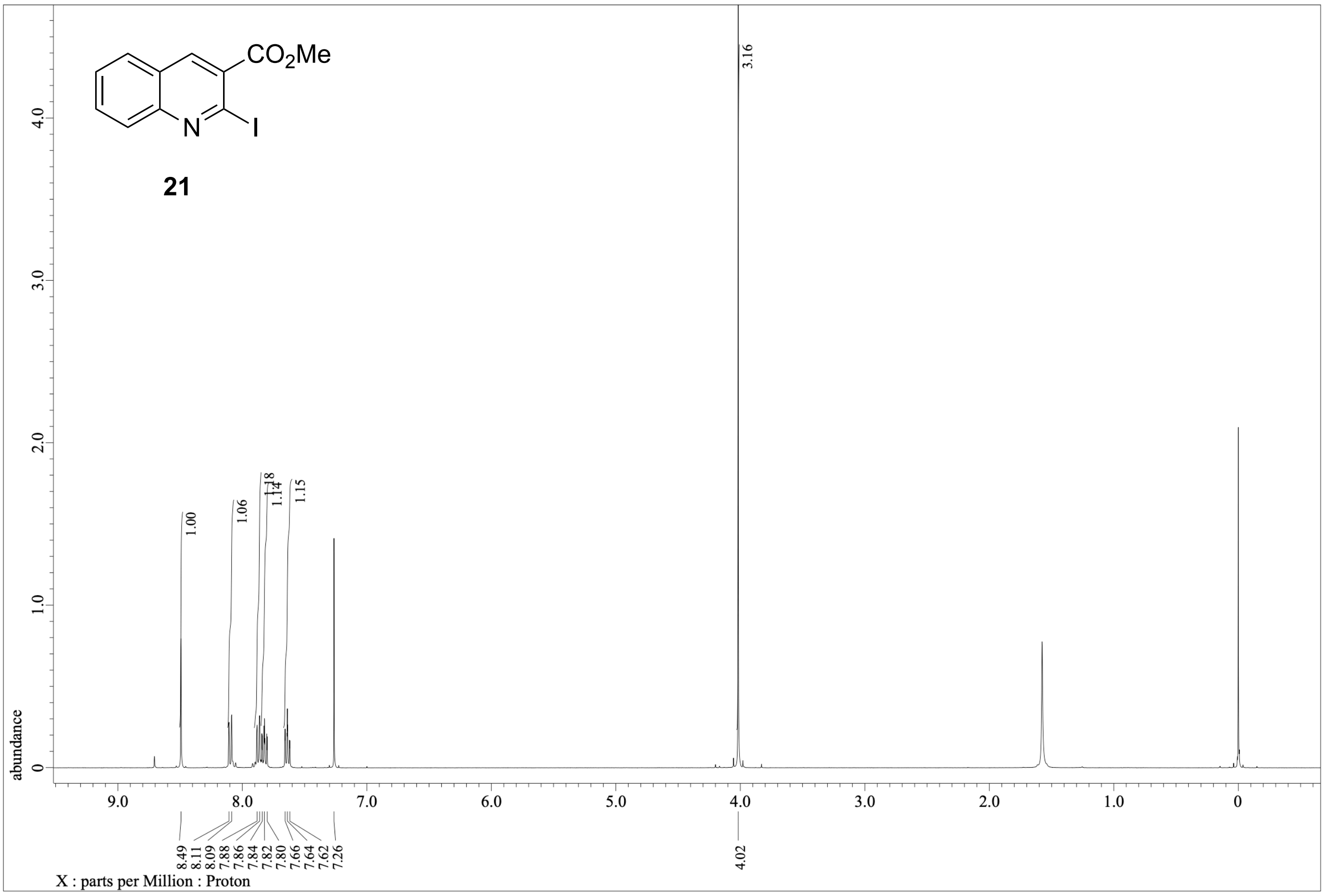

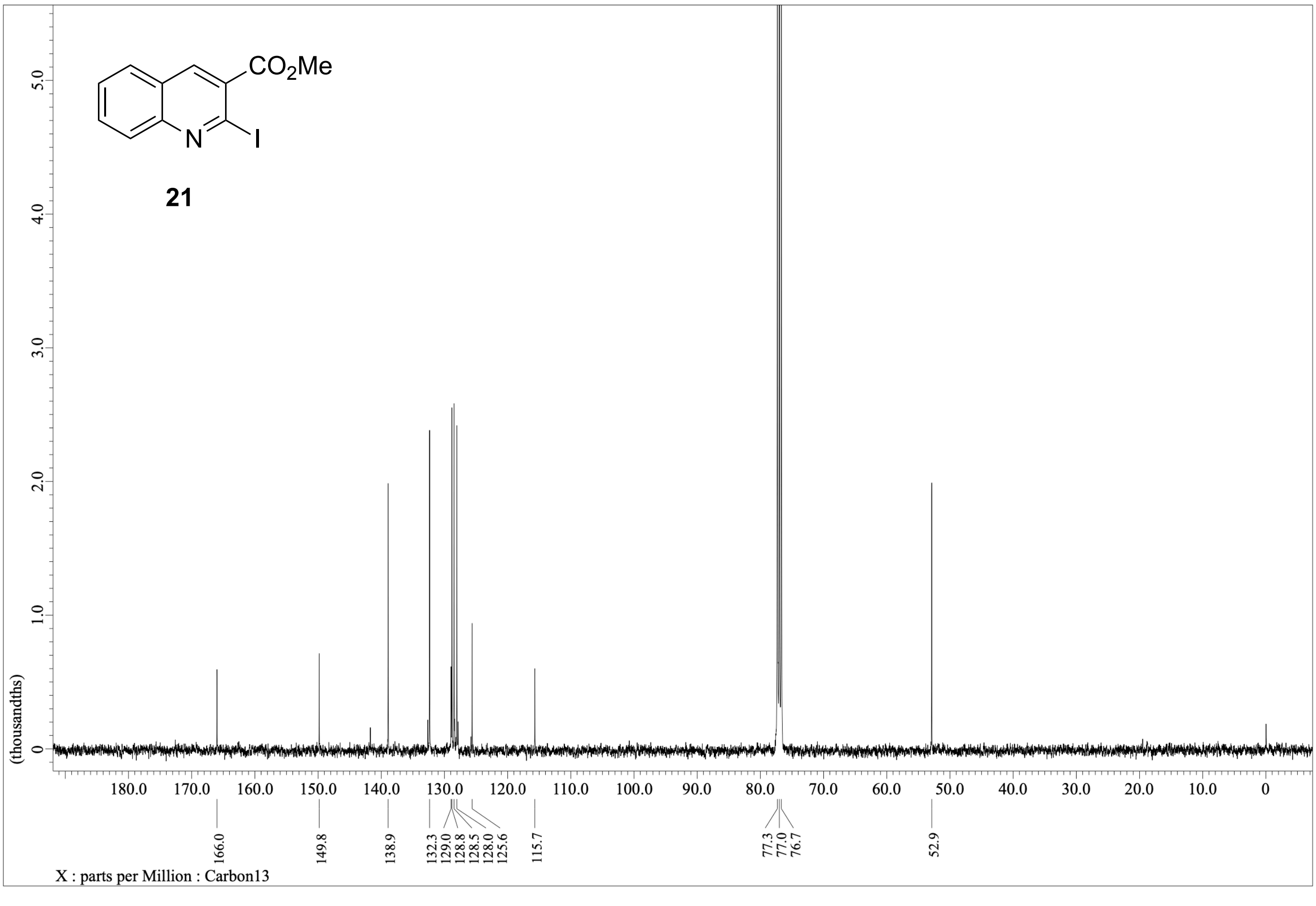

2-Iodoquinolin-3-ylmethanol (22)

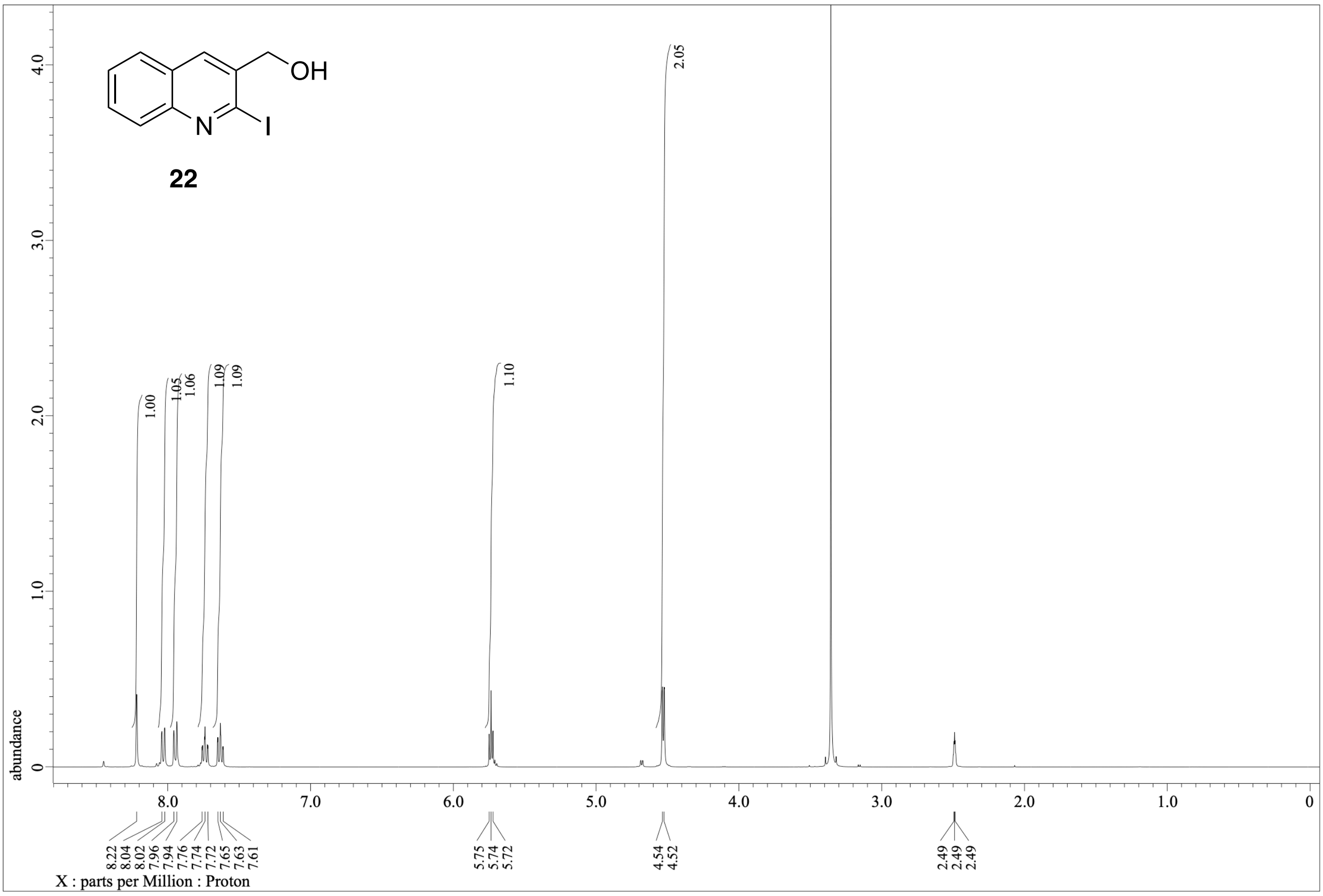

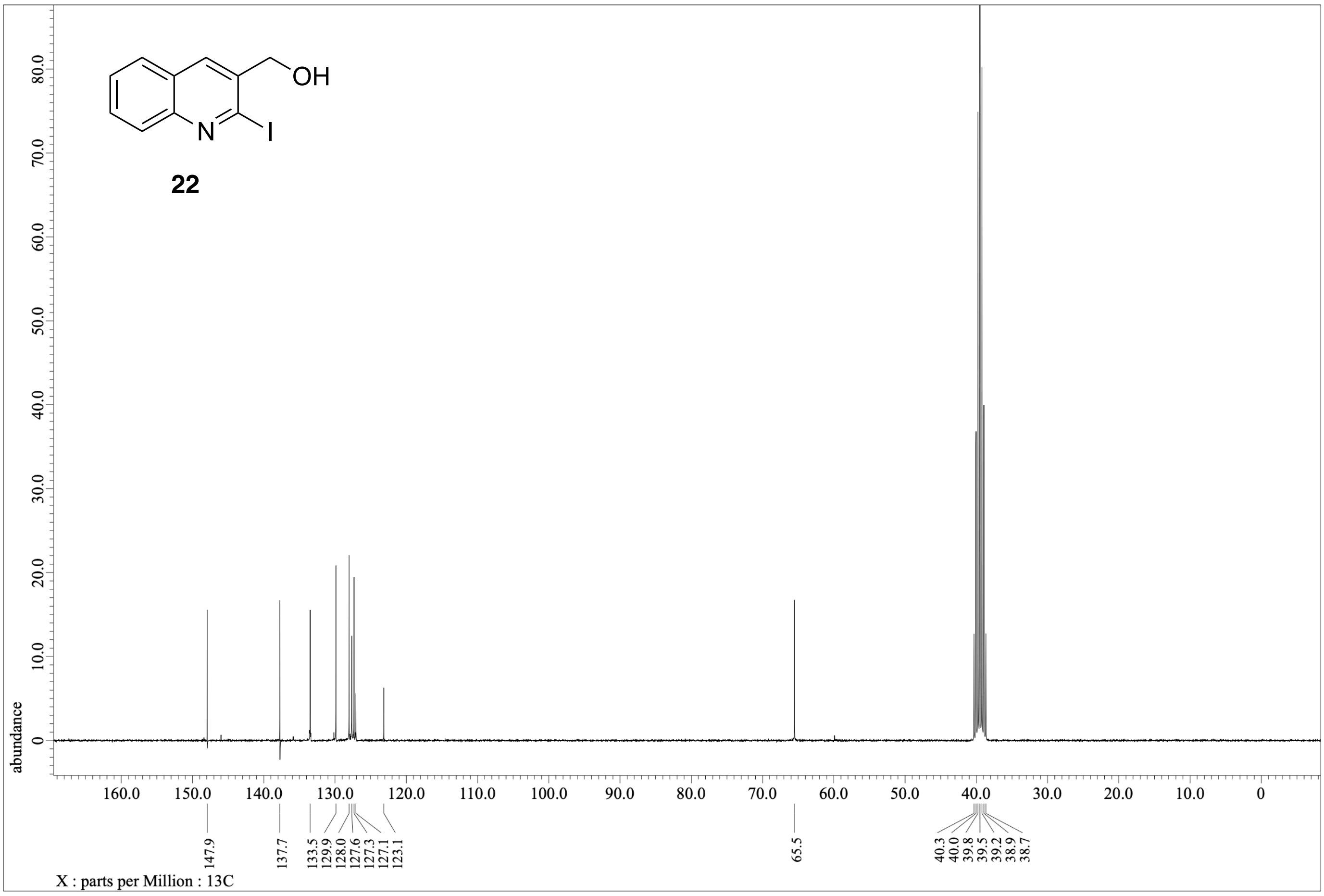

2-Iodo-3-methoxymethylquinoline (23)

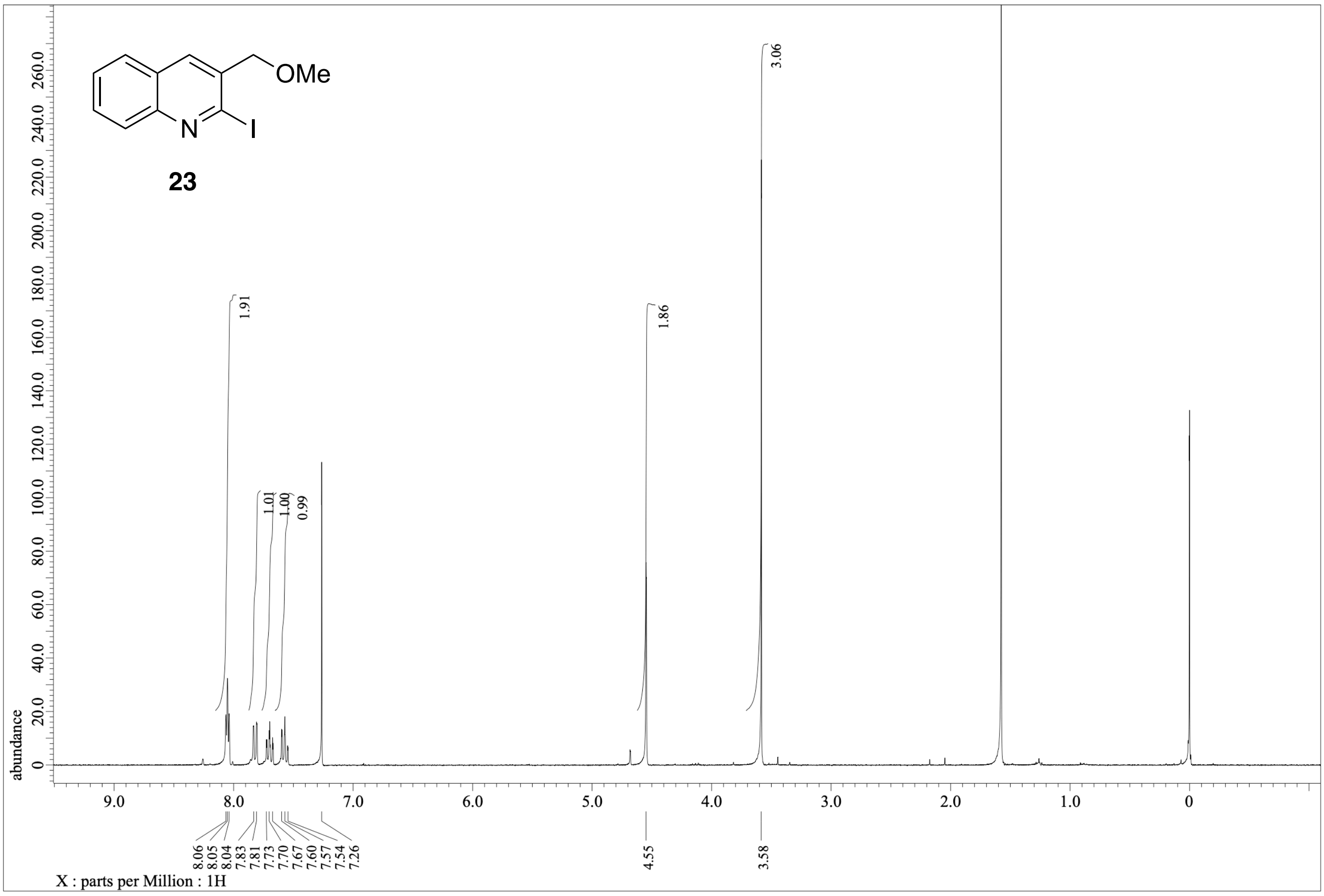

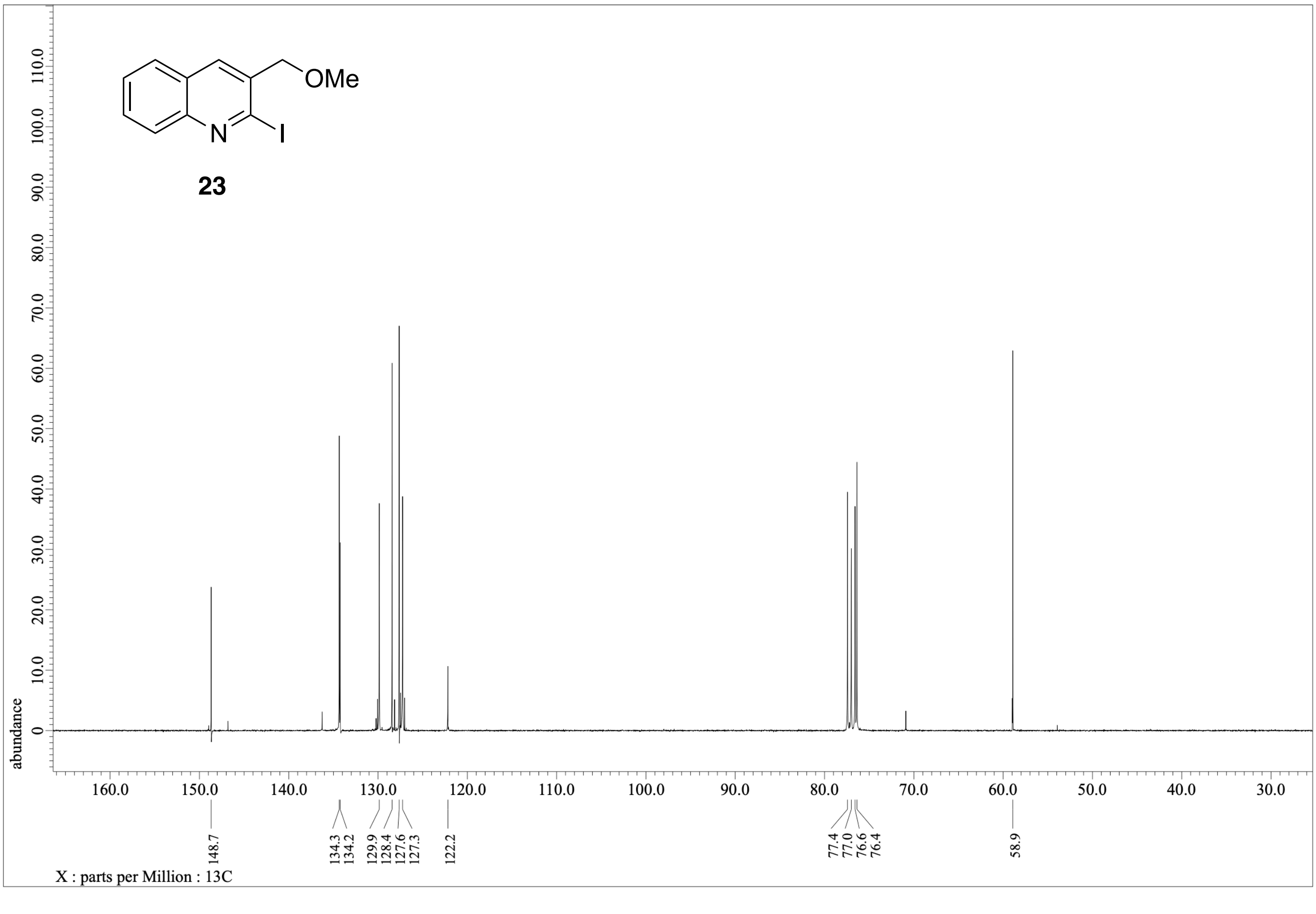

Methyl 2-(2-formylphenyl)ethynylquinoline-3-carboxylate (24a)

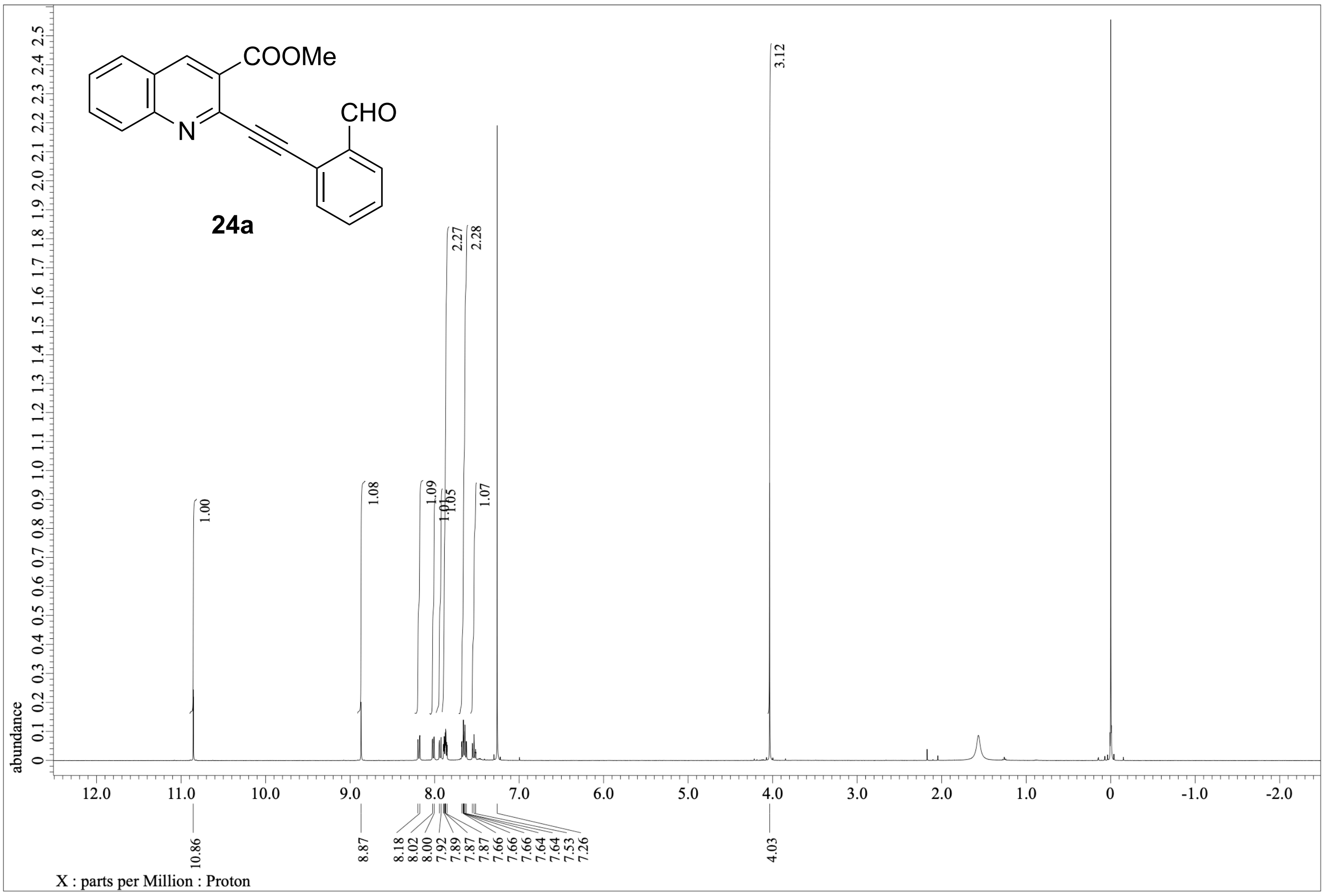

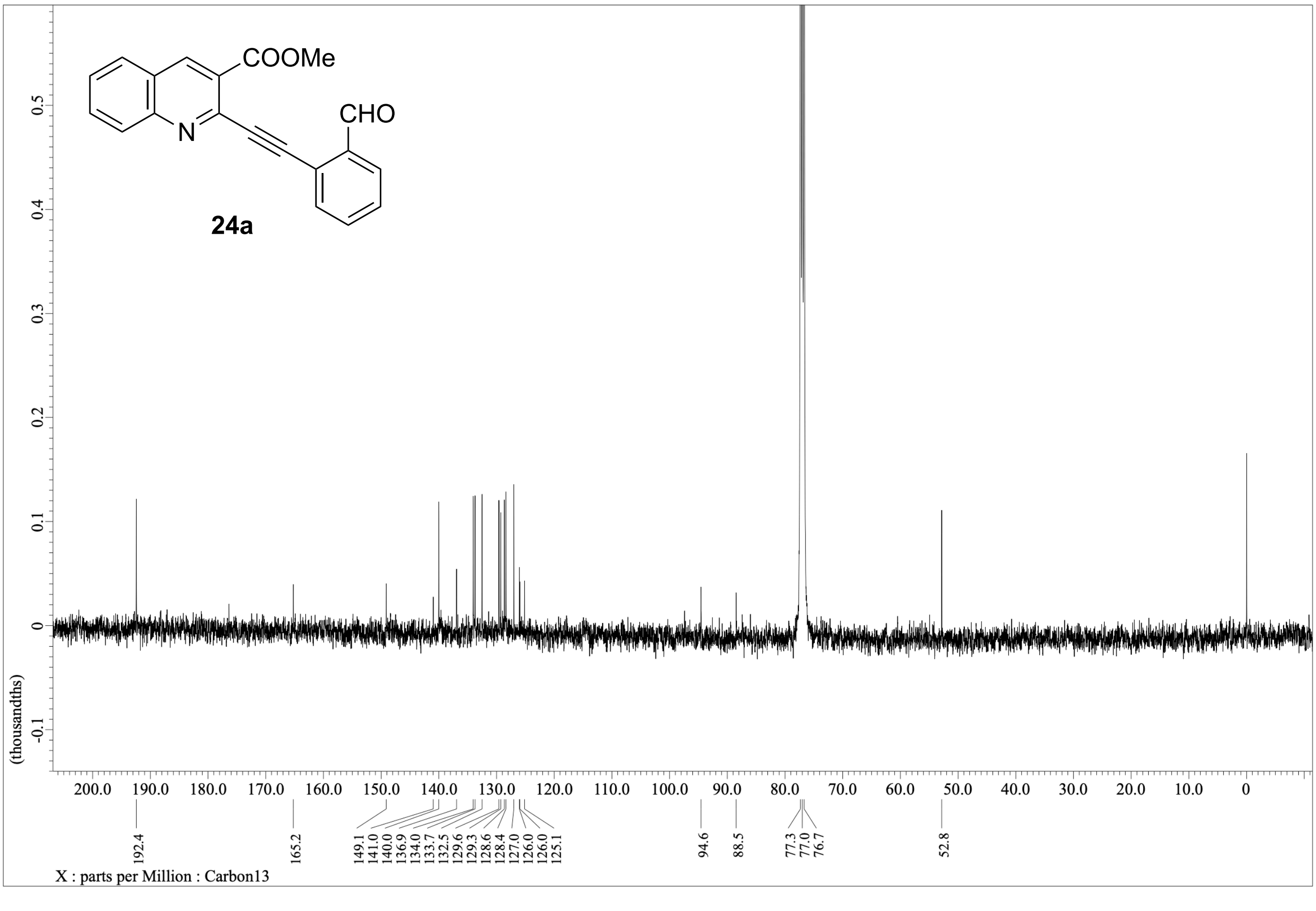

2-(3-Methylquinolin-2-yl)ethynylbenzaldehyde (24b)

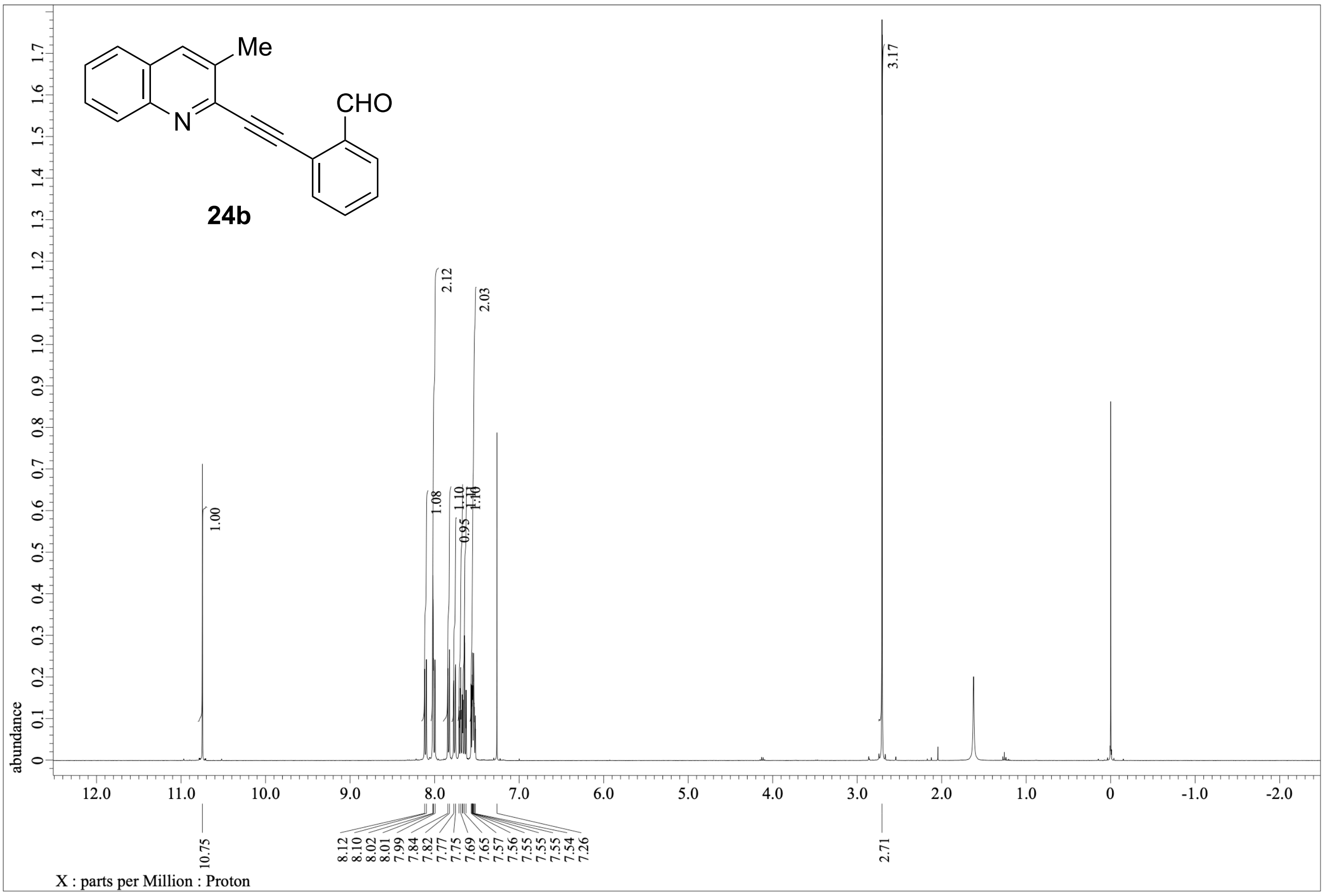

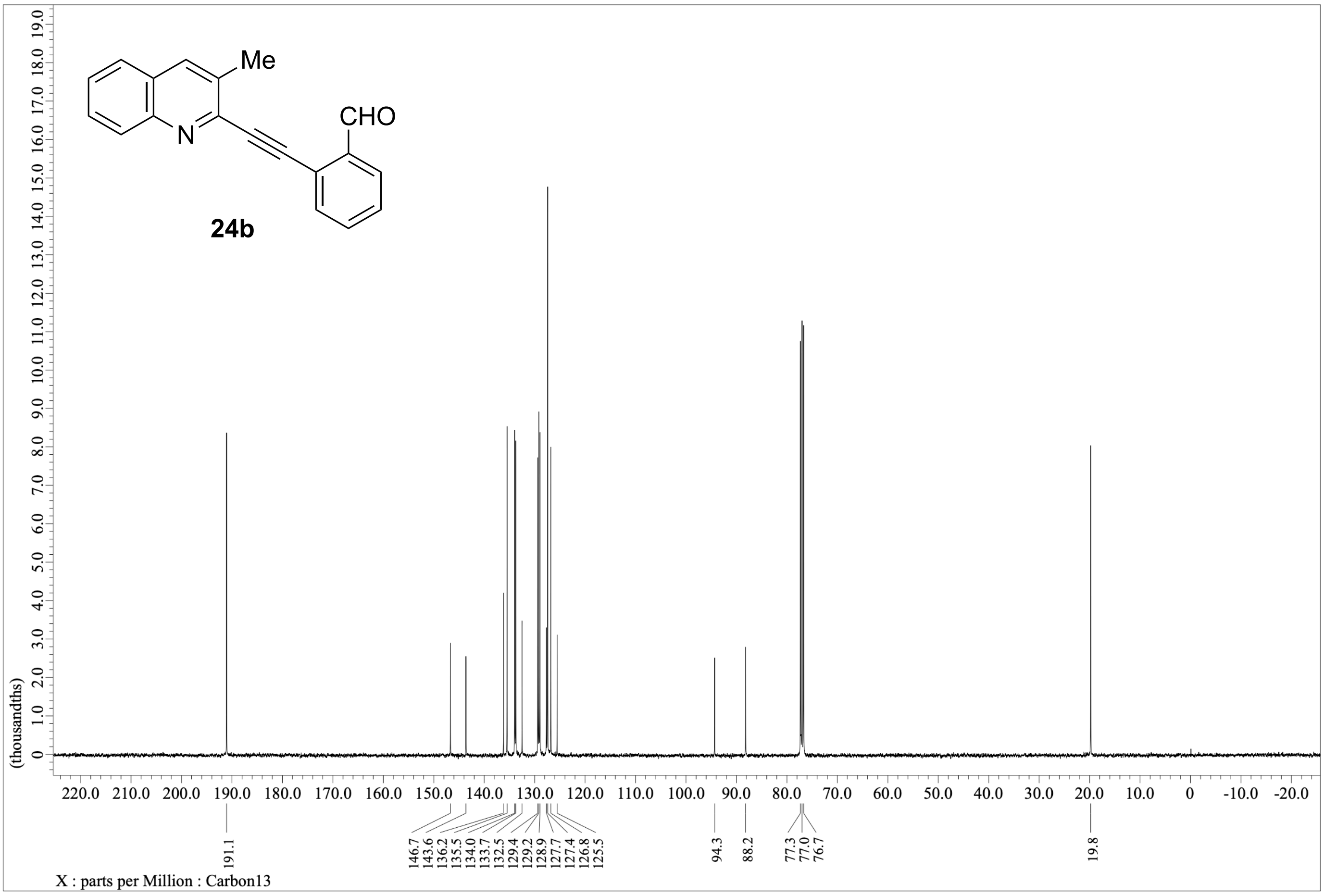

2-(3-Methoxymethylquinolin-2-yl)ethynylbenzaldehyde (24c)

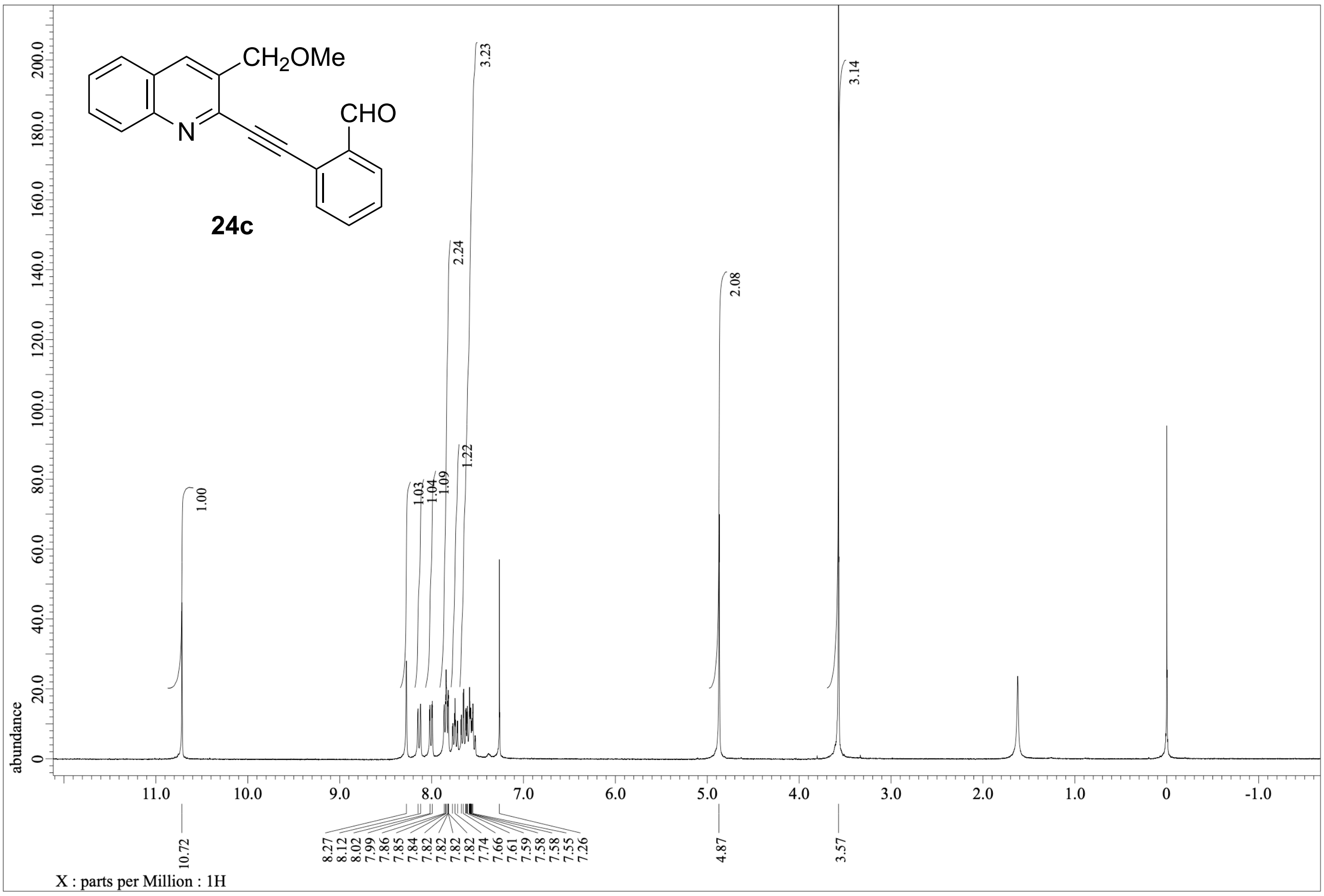

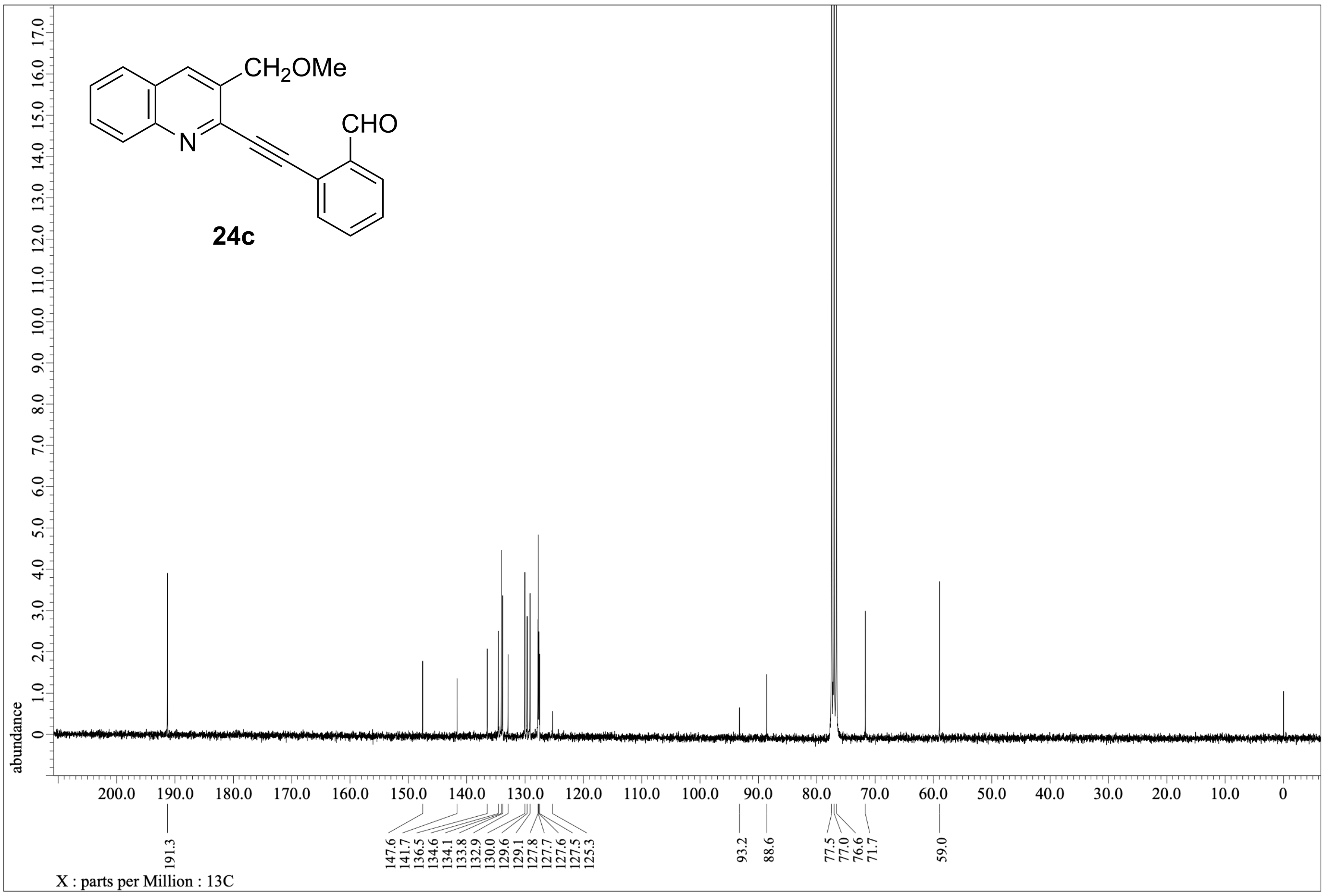

Methyl 2-(2-hydroxyiminomethylphenyl)ethynylquinoline-3-carboxylate (25a)

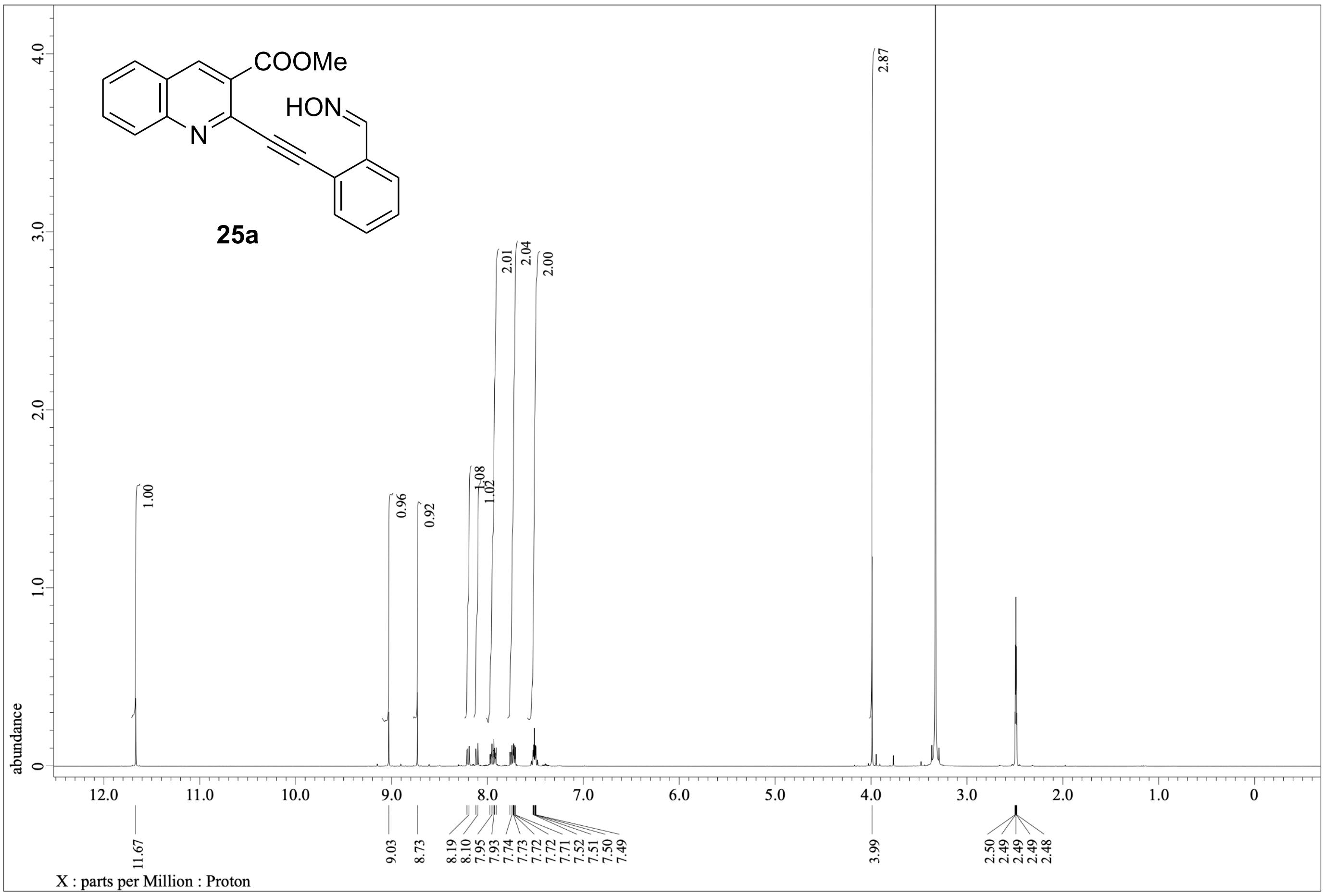

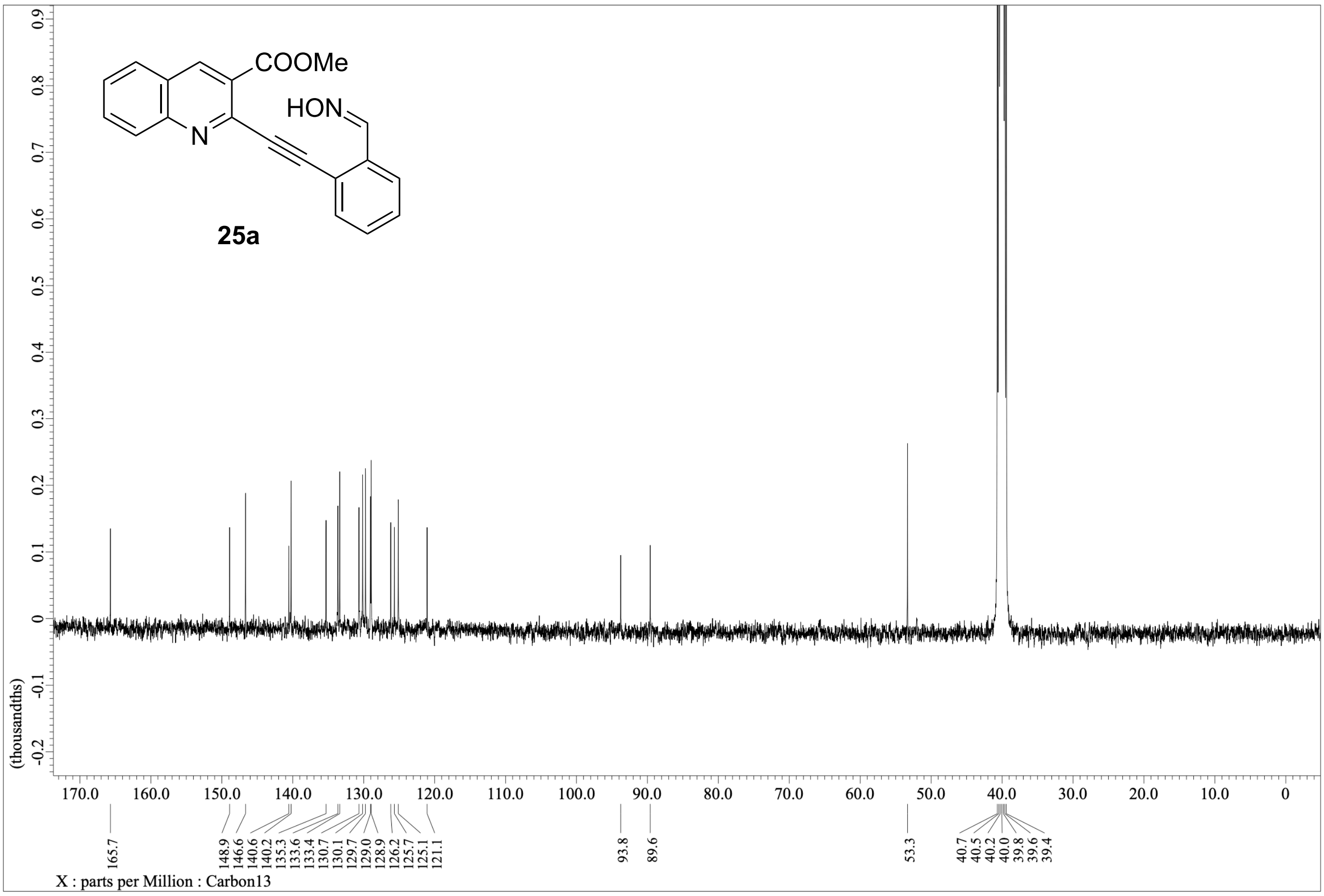

2-(3-Methylquinolin-2-yl)ethynylbenzaldehyde oxime (25b)

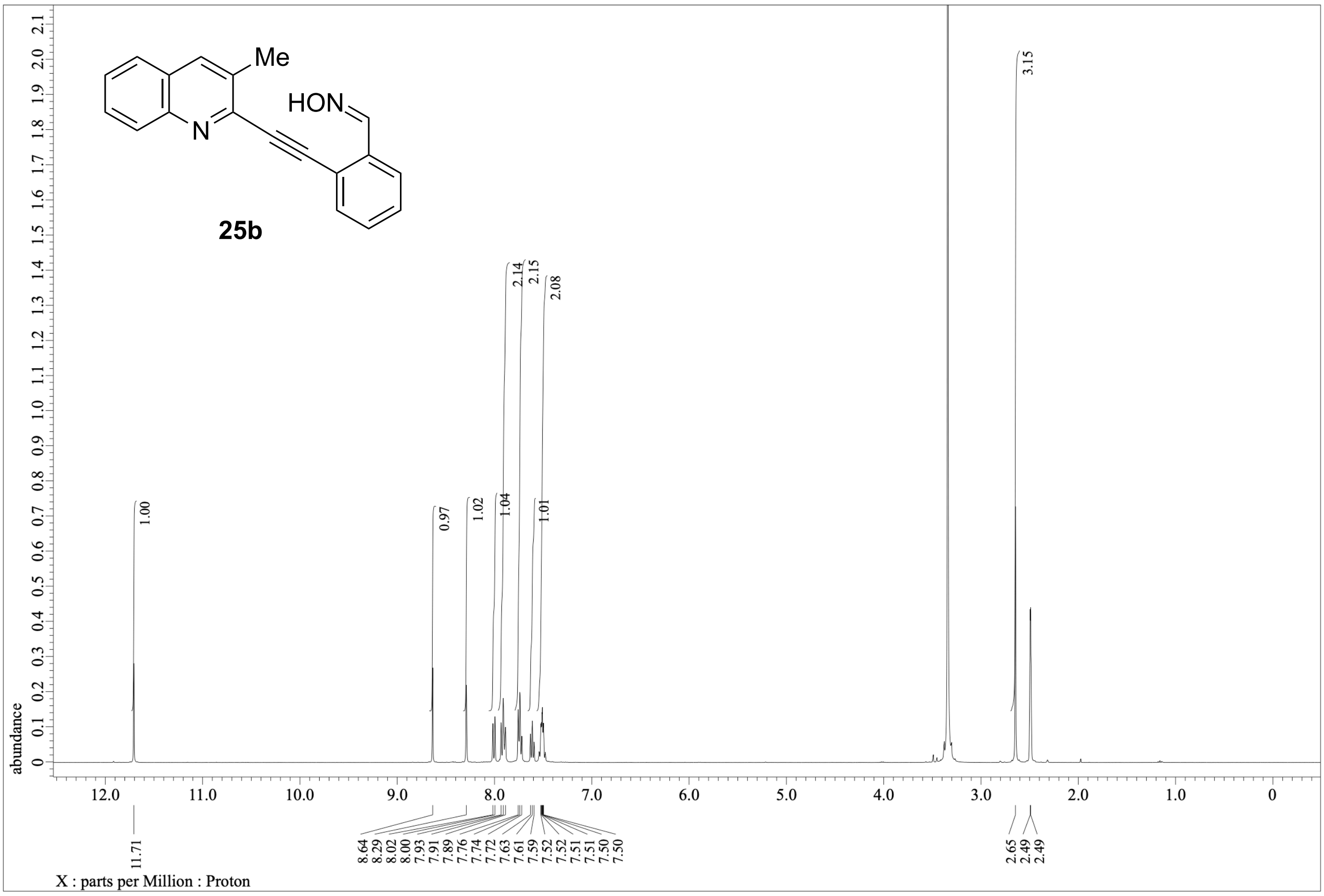

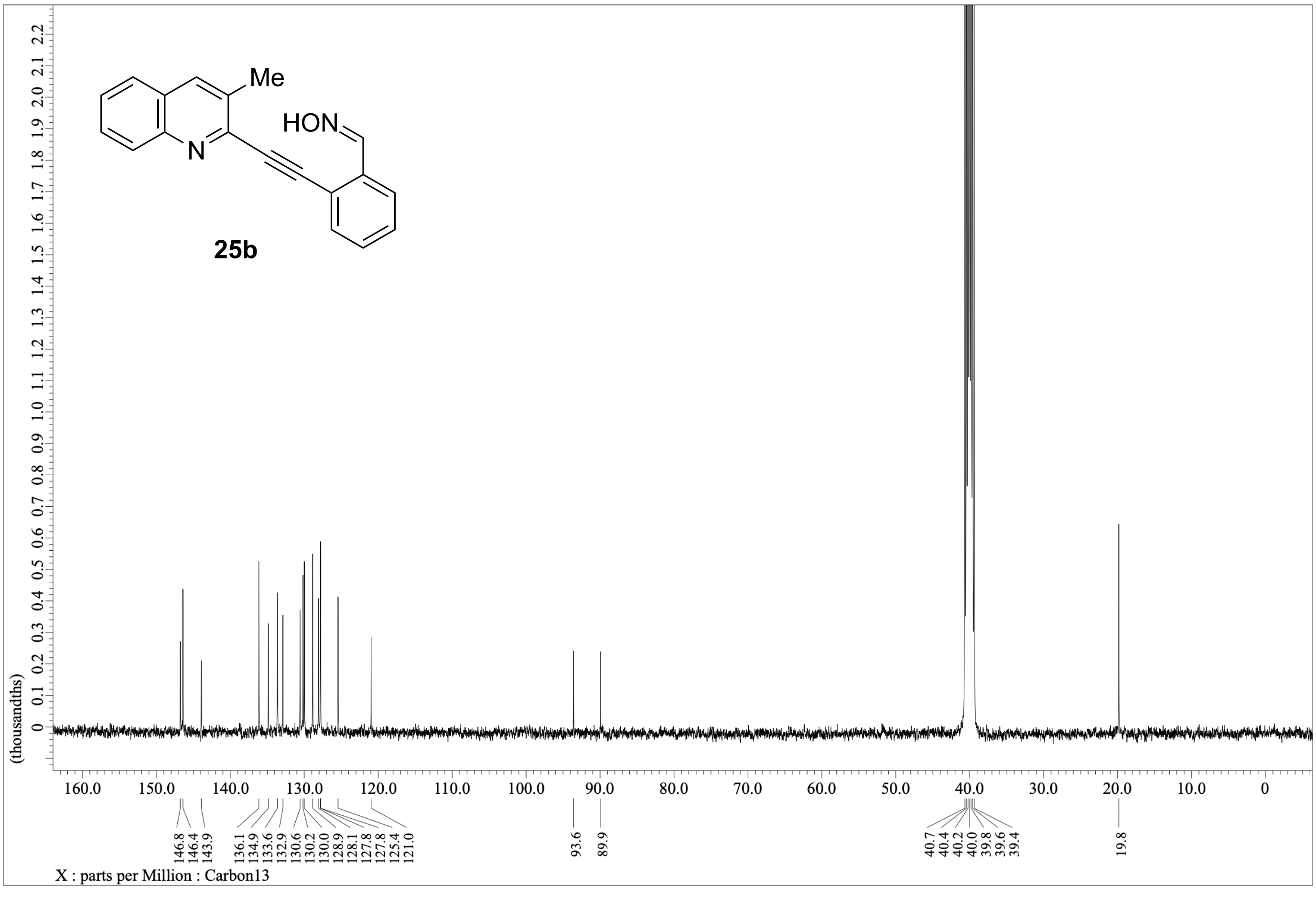

2-(3-Methoxymethylquinolin-2-yl)ethynylbenzaldehyde oxime (25c)

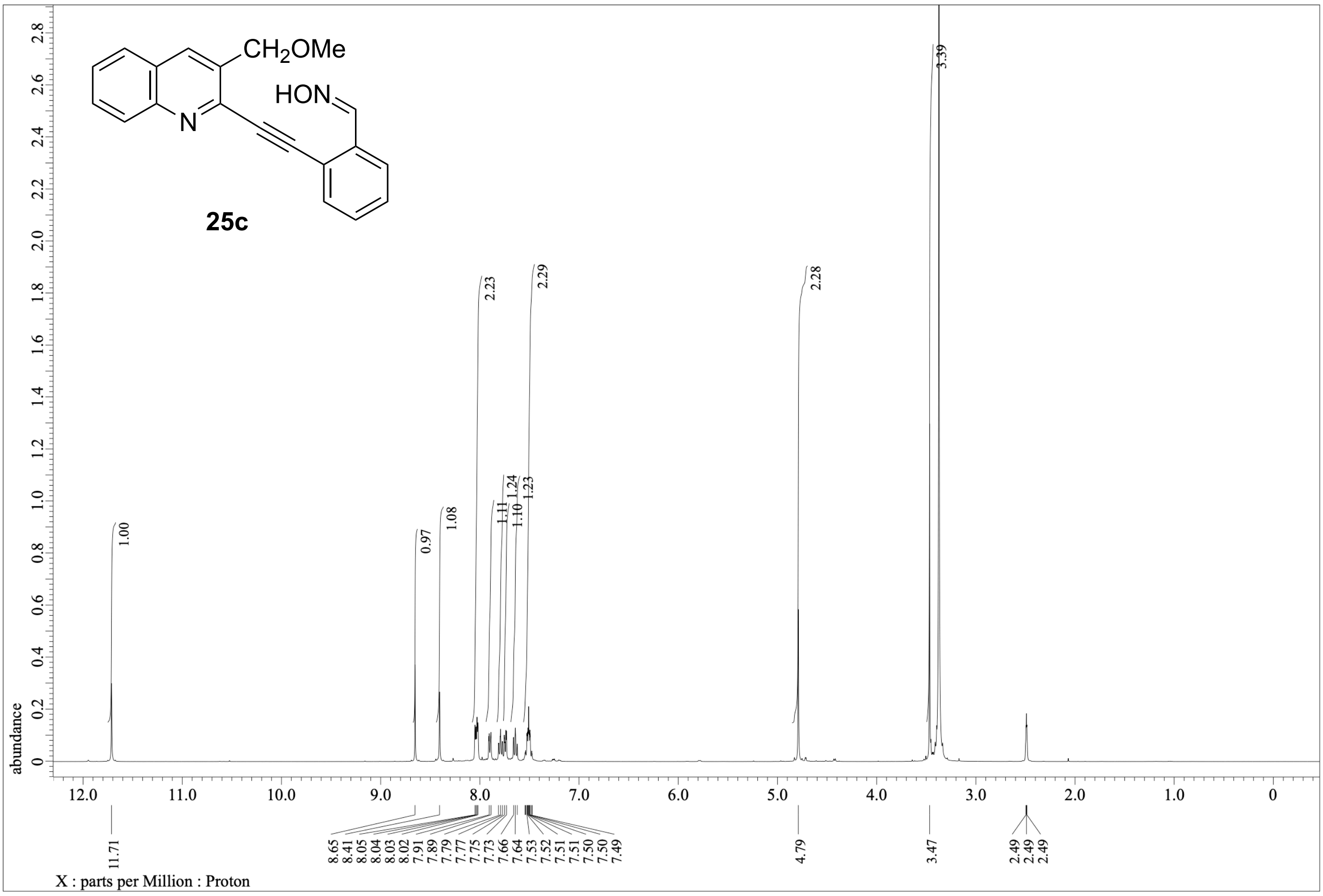

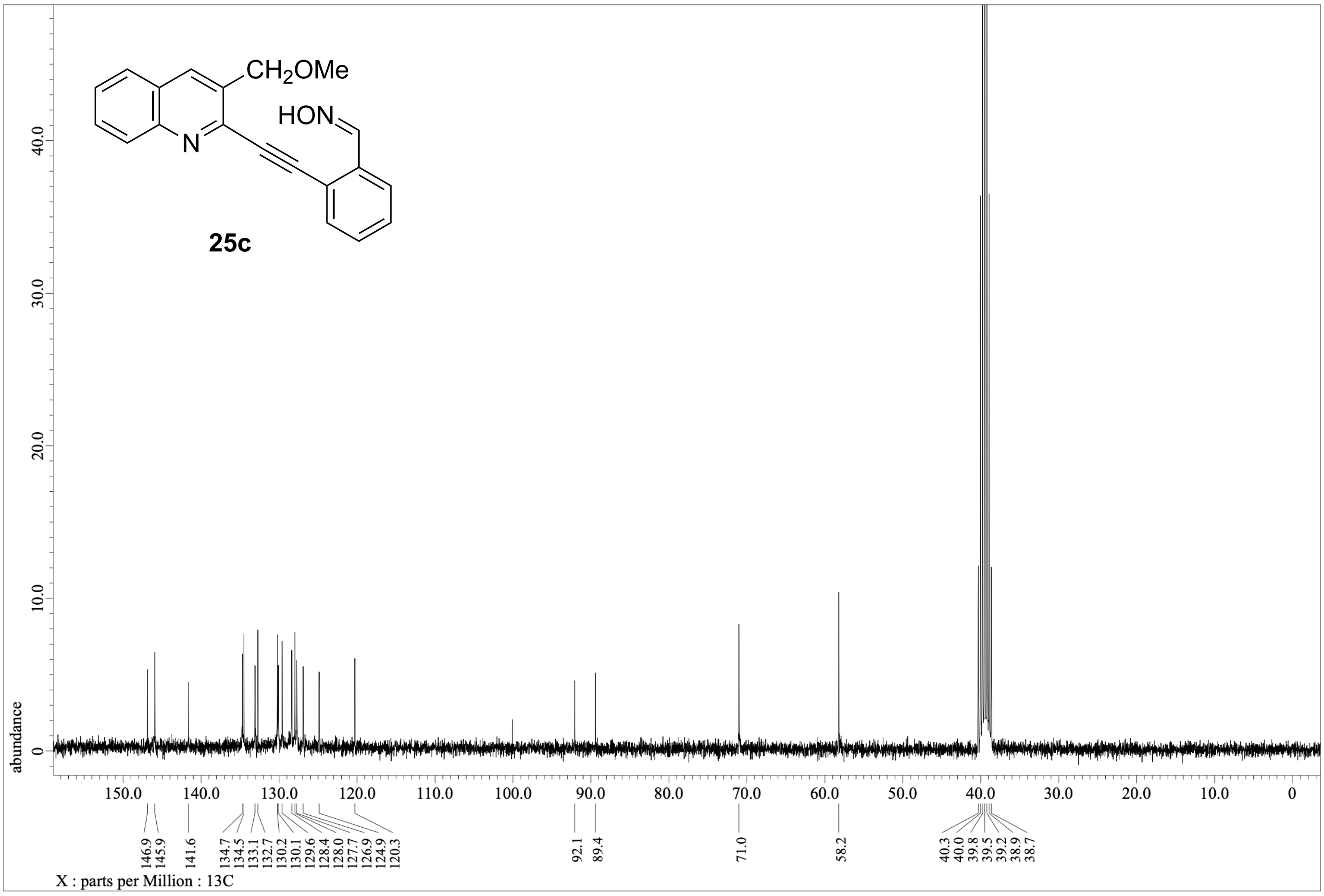

3-(3-Methylquinolin-2-yl)isoquinolin-1-one (27b)

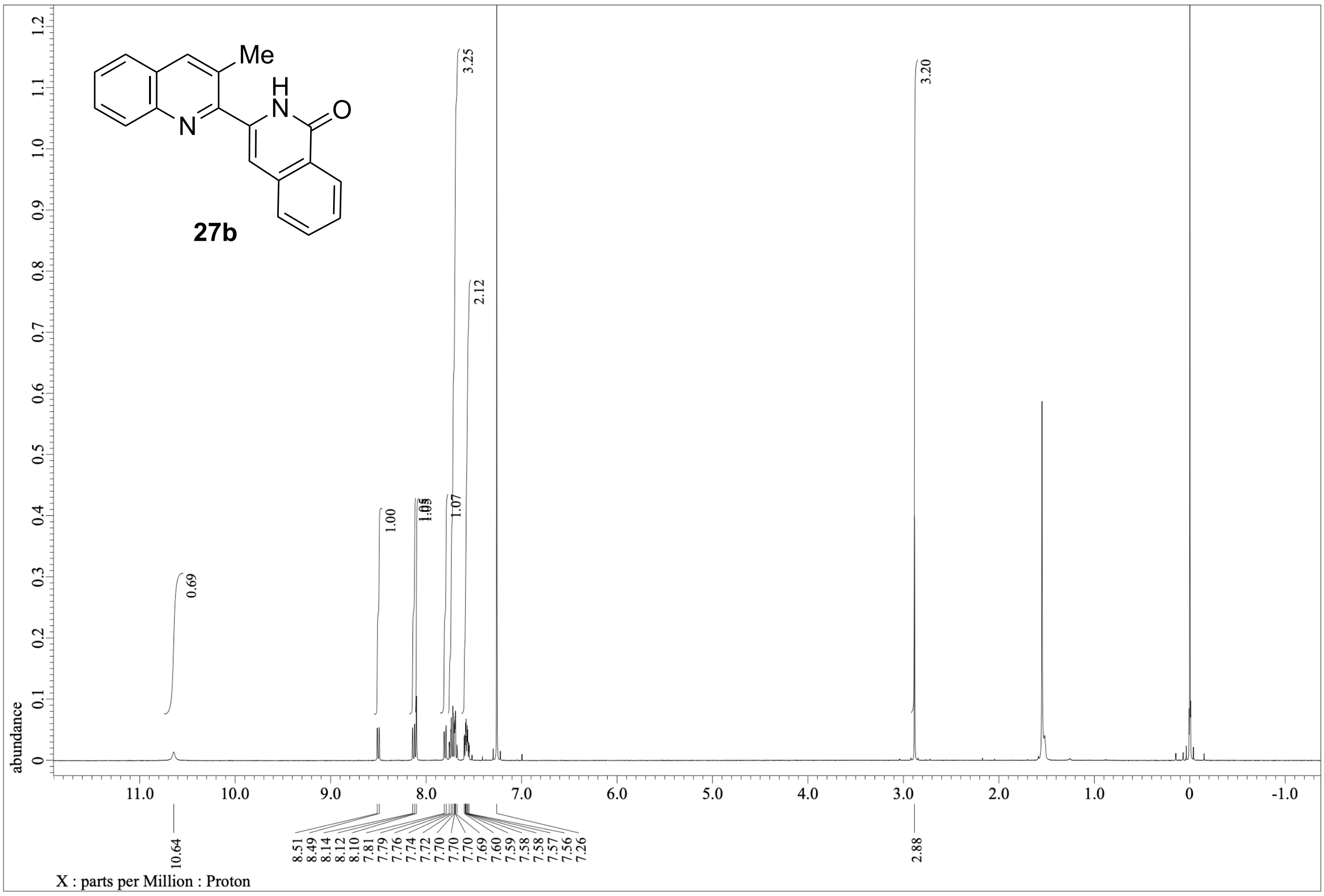

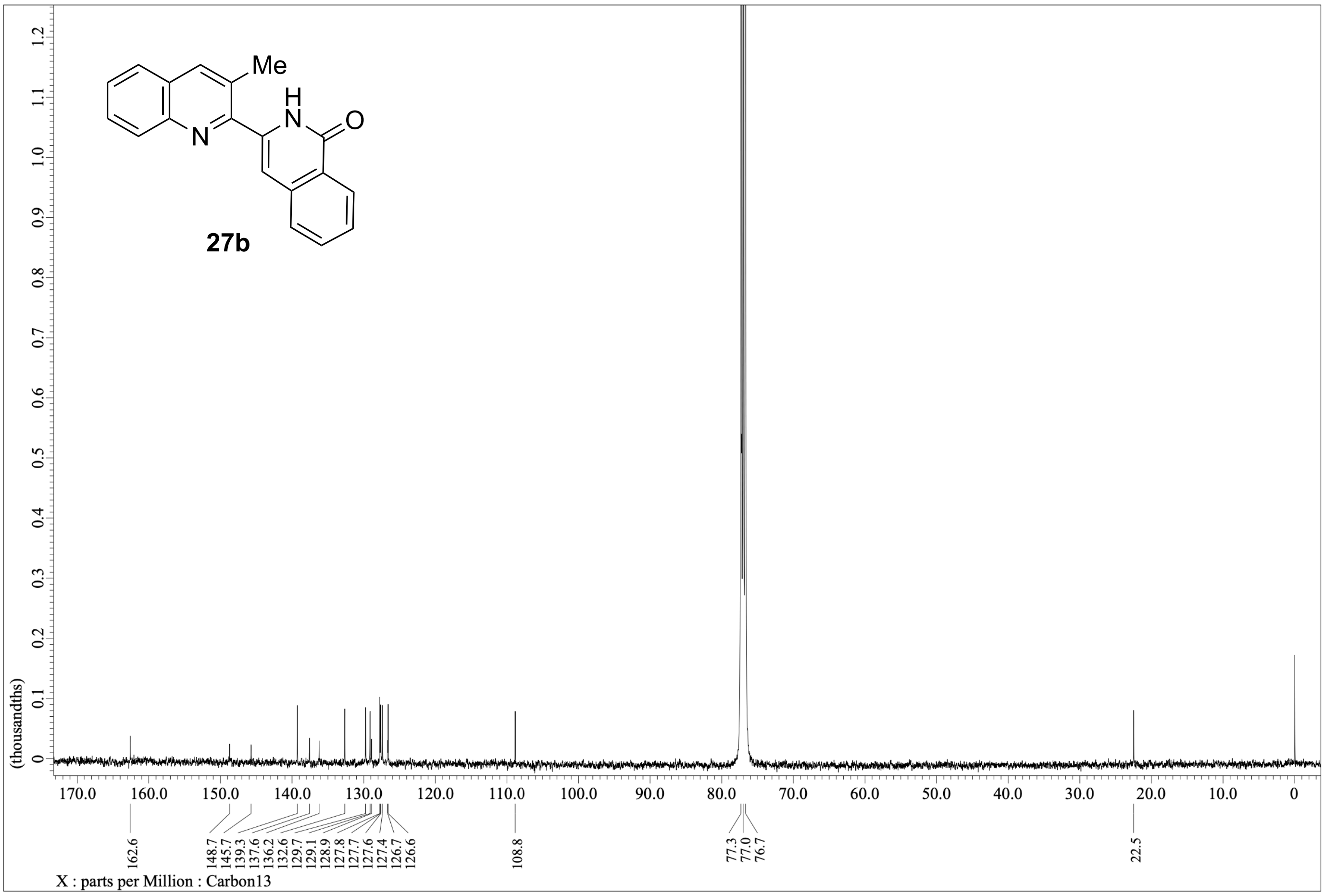

4-Acetoxy-3-(3-methylquinolin-2-yl)isoquinoline (28b)

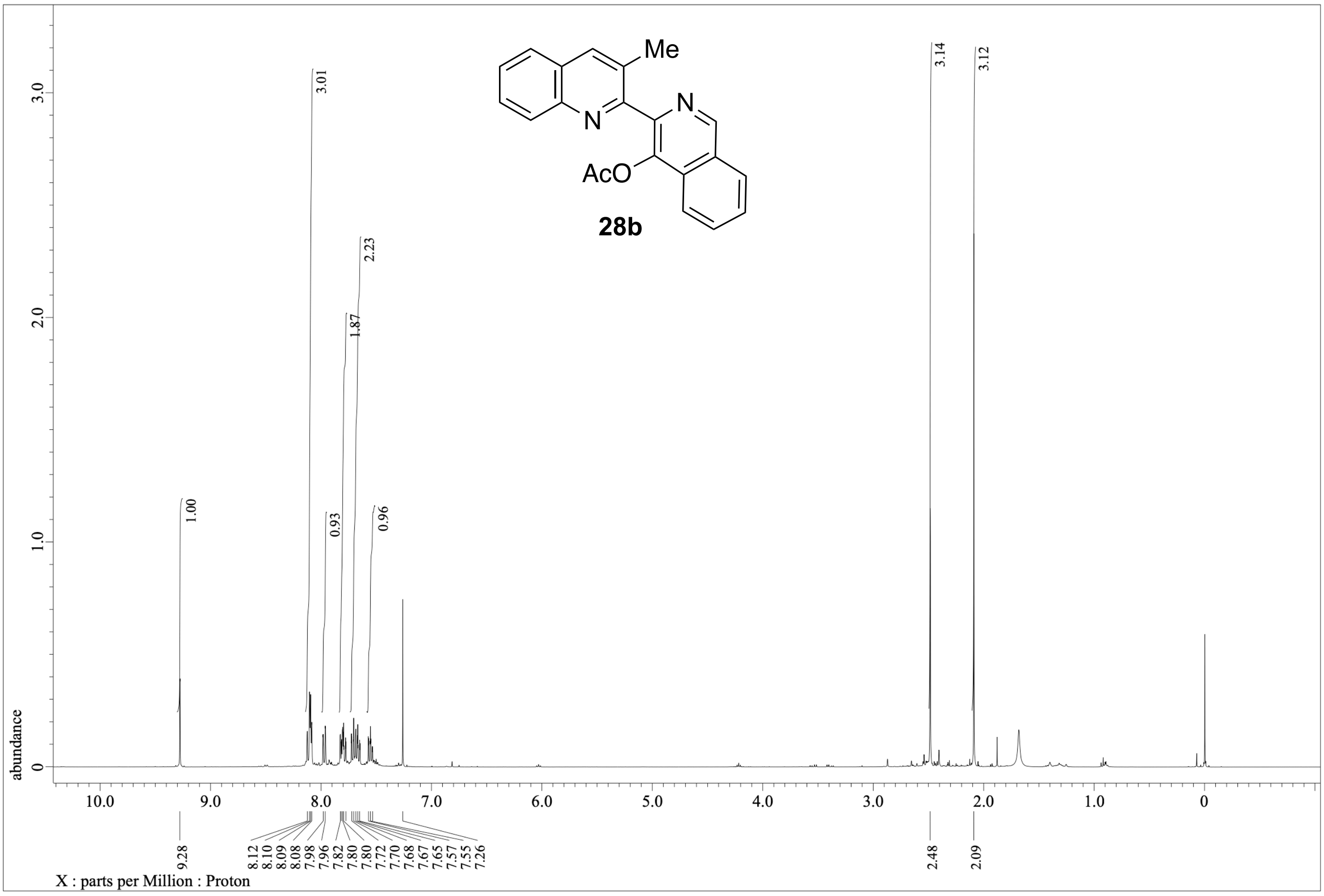

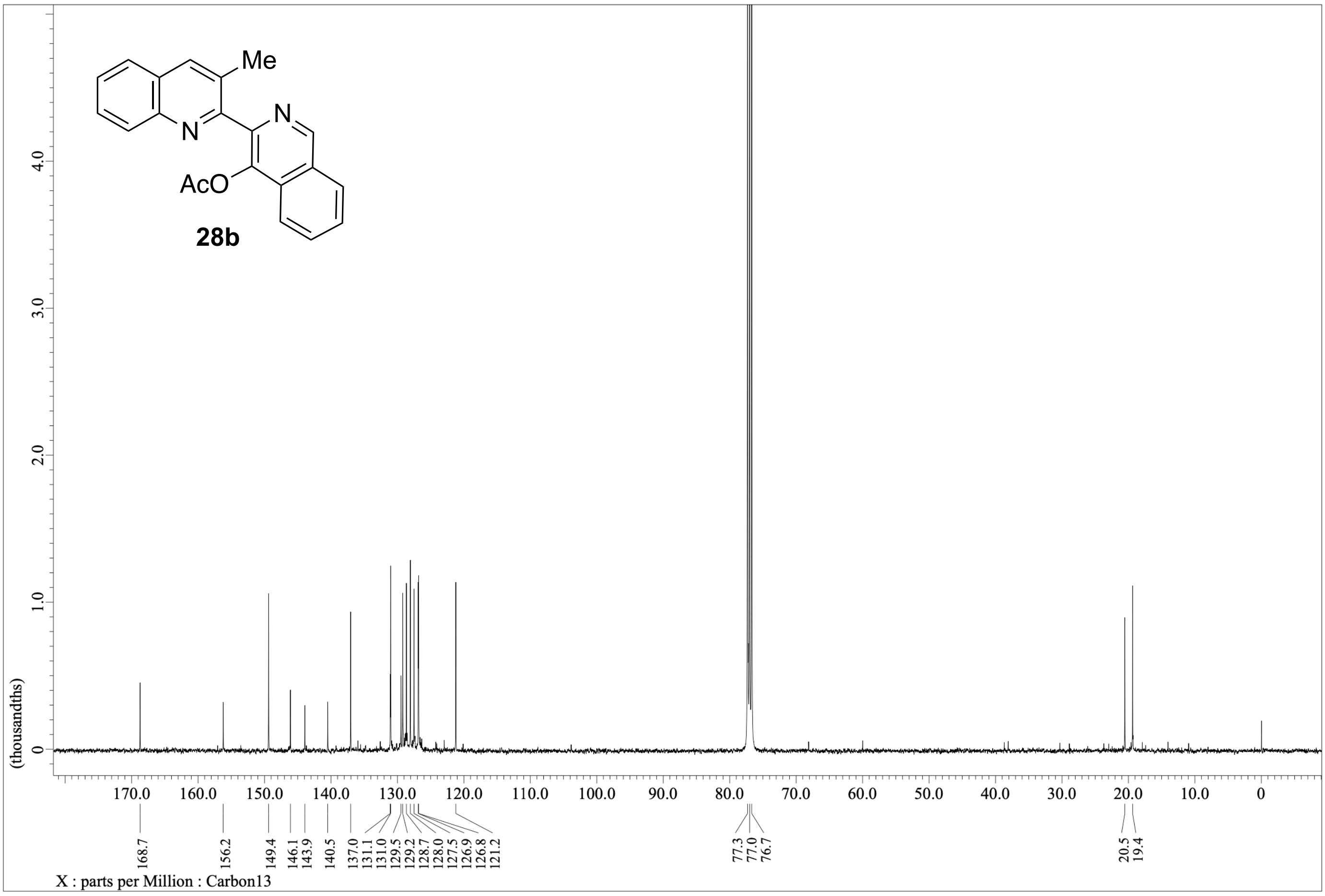

3-(3-Methoxymethylquinolin-2-yl)isoquinoline *N*-oxide (26c)

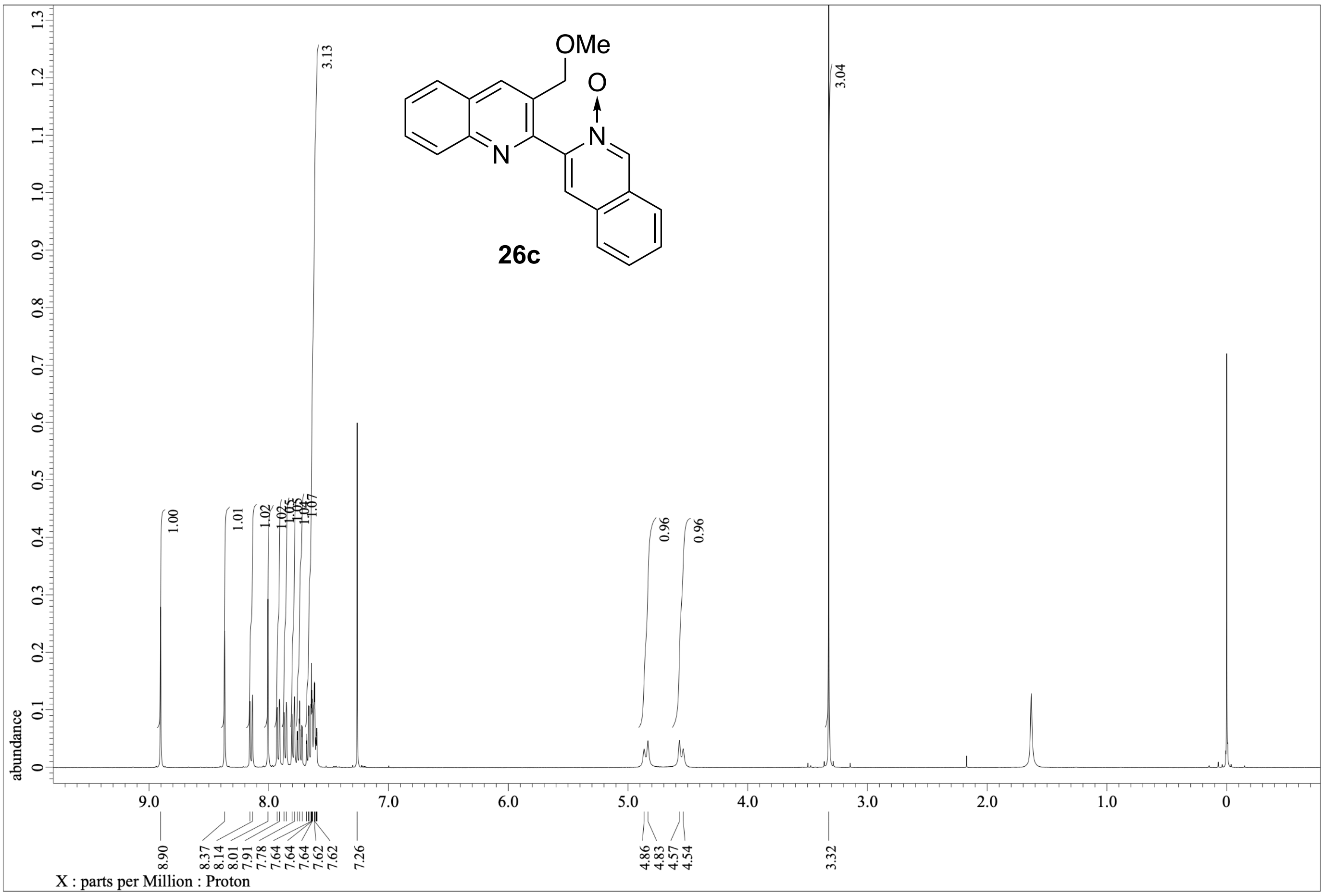

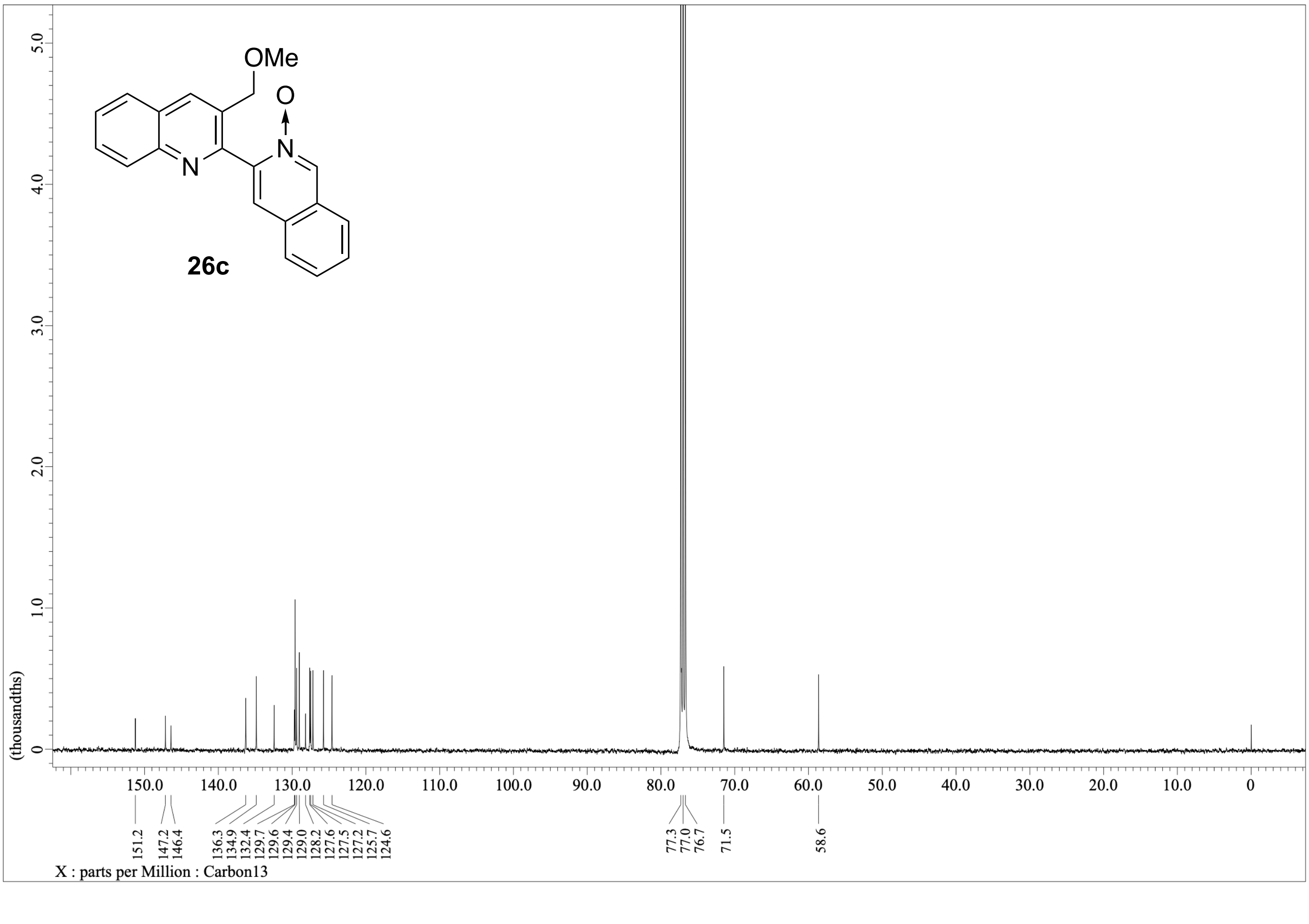

3-(3-Methoxymethylquinolin-2-yl)isoquinolin-1-one (27c)

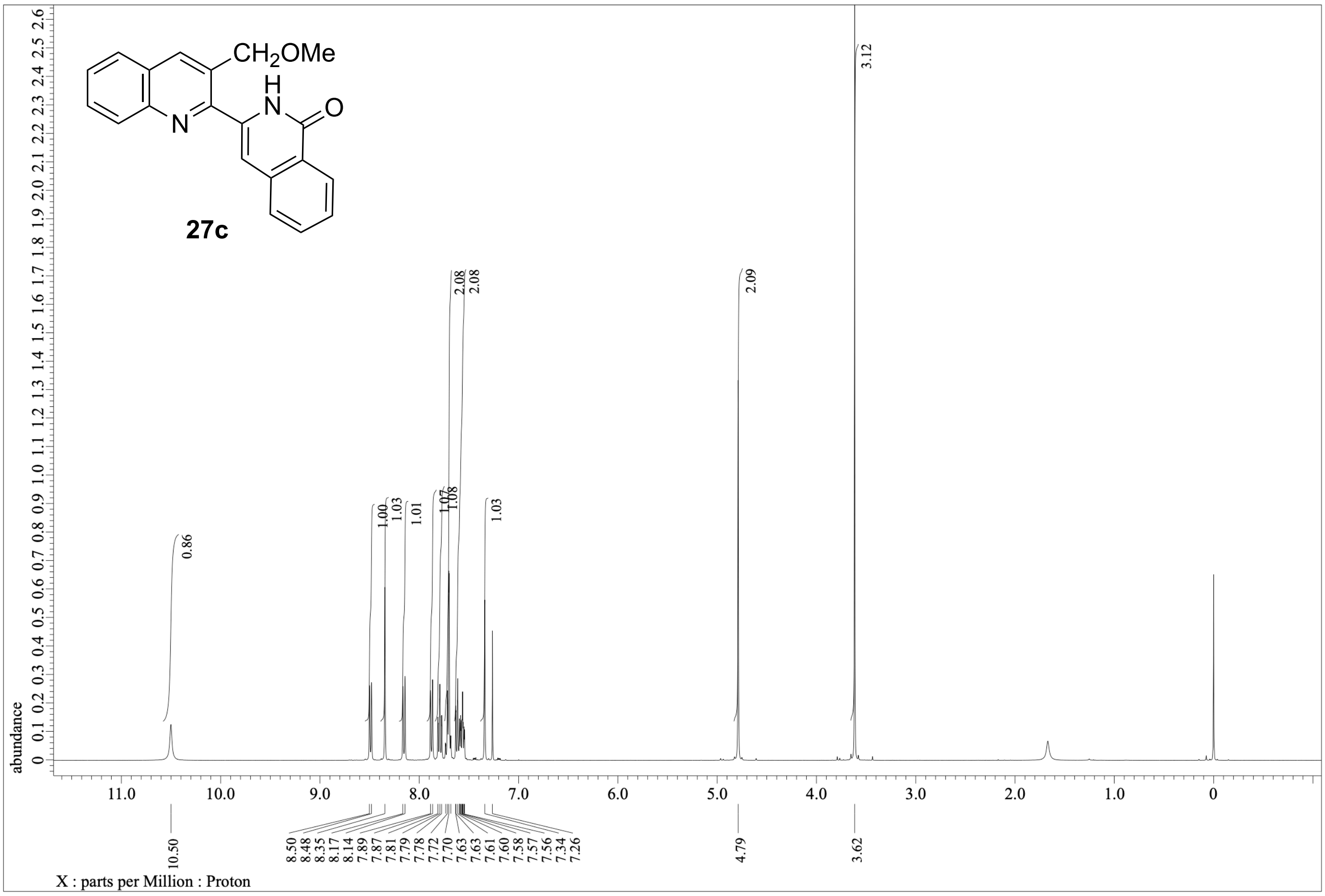

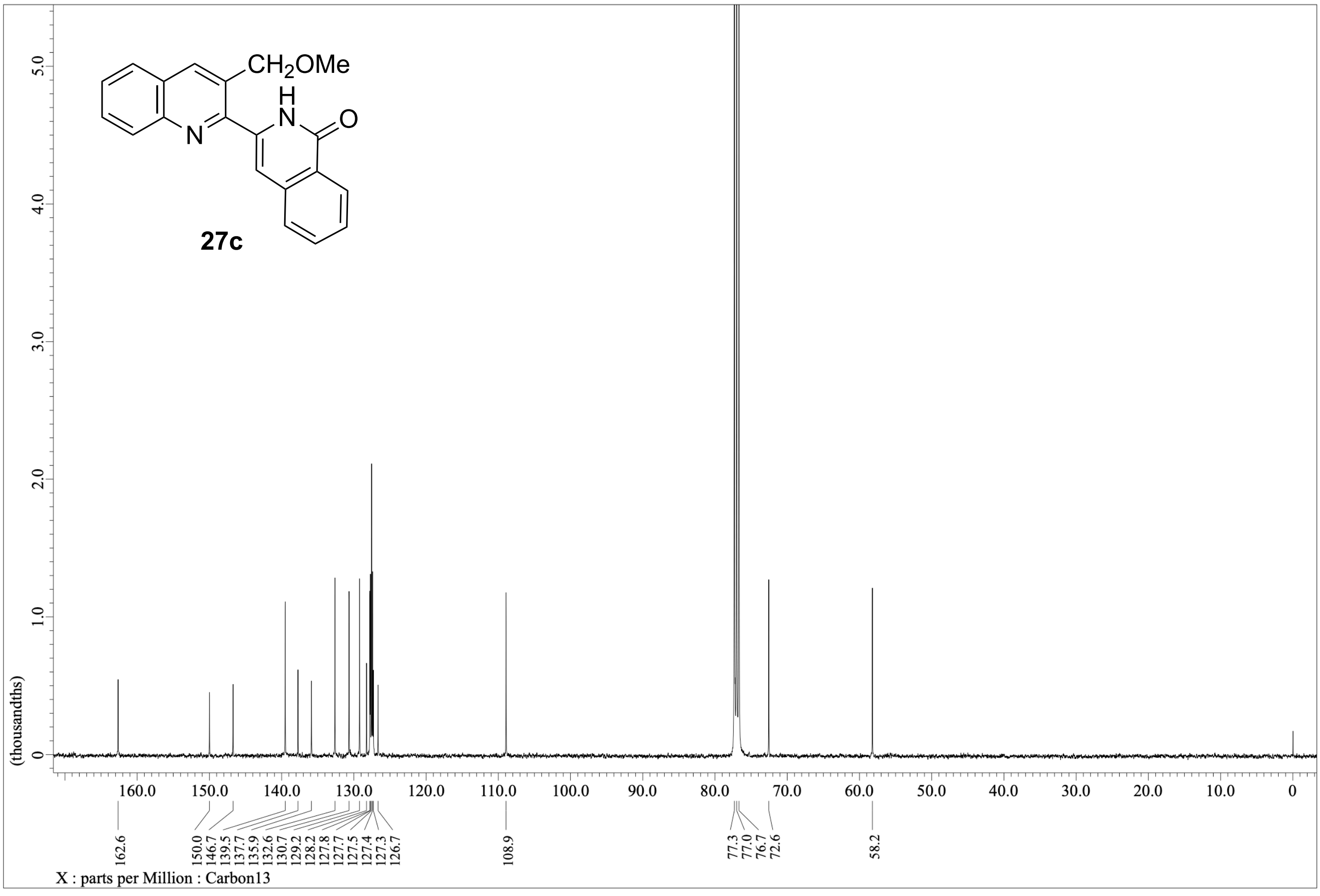

4-Acetoxy-3-(3-methoxymethylquinolin-2-yl)isoquinoline (28c)

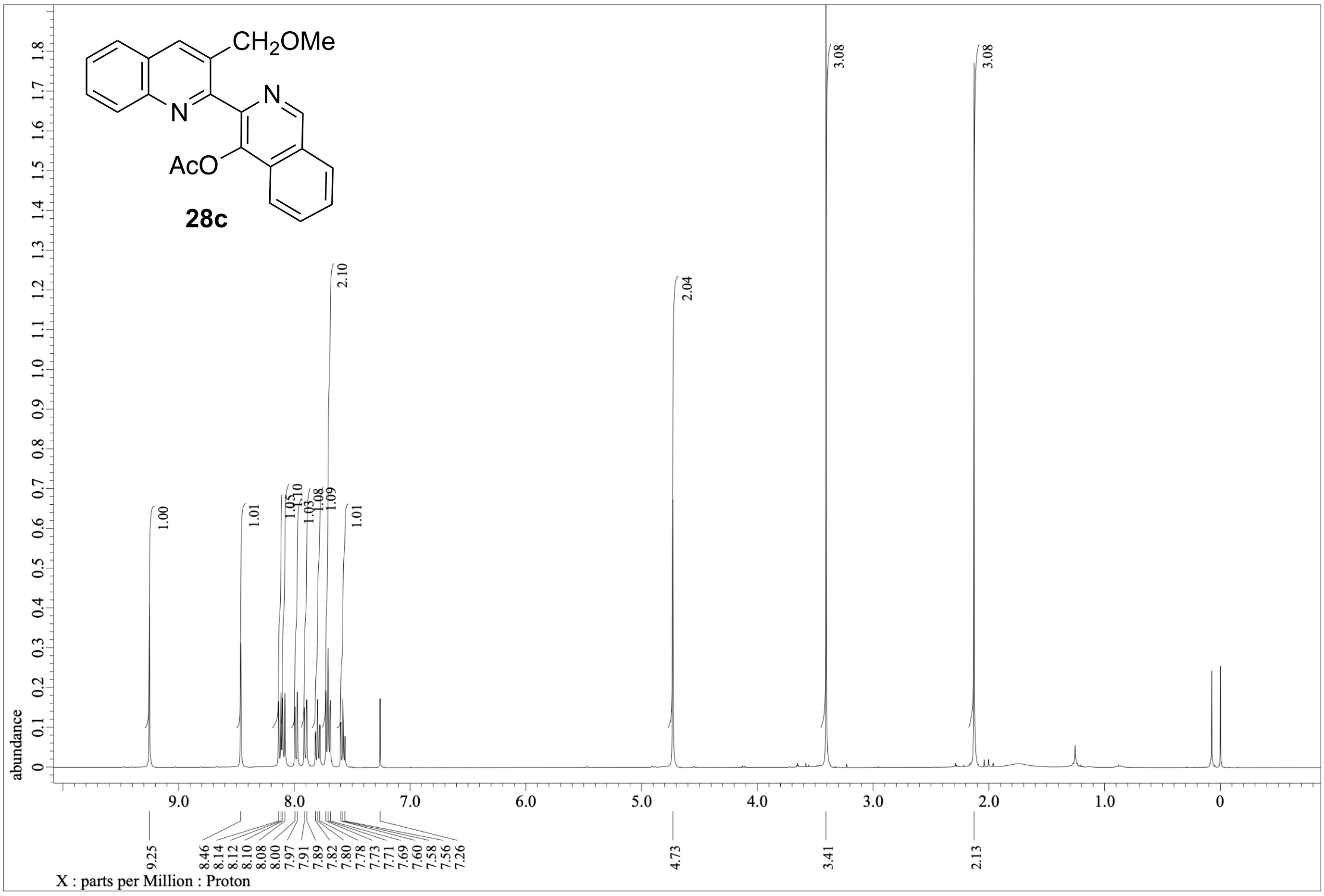

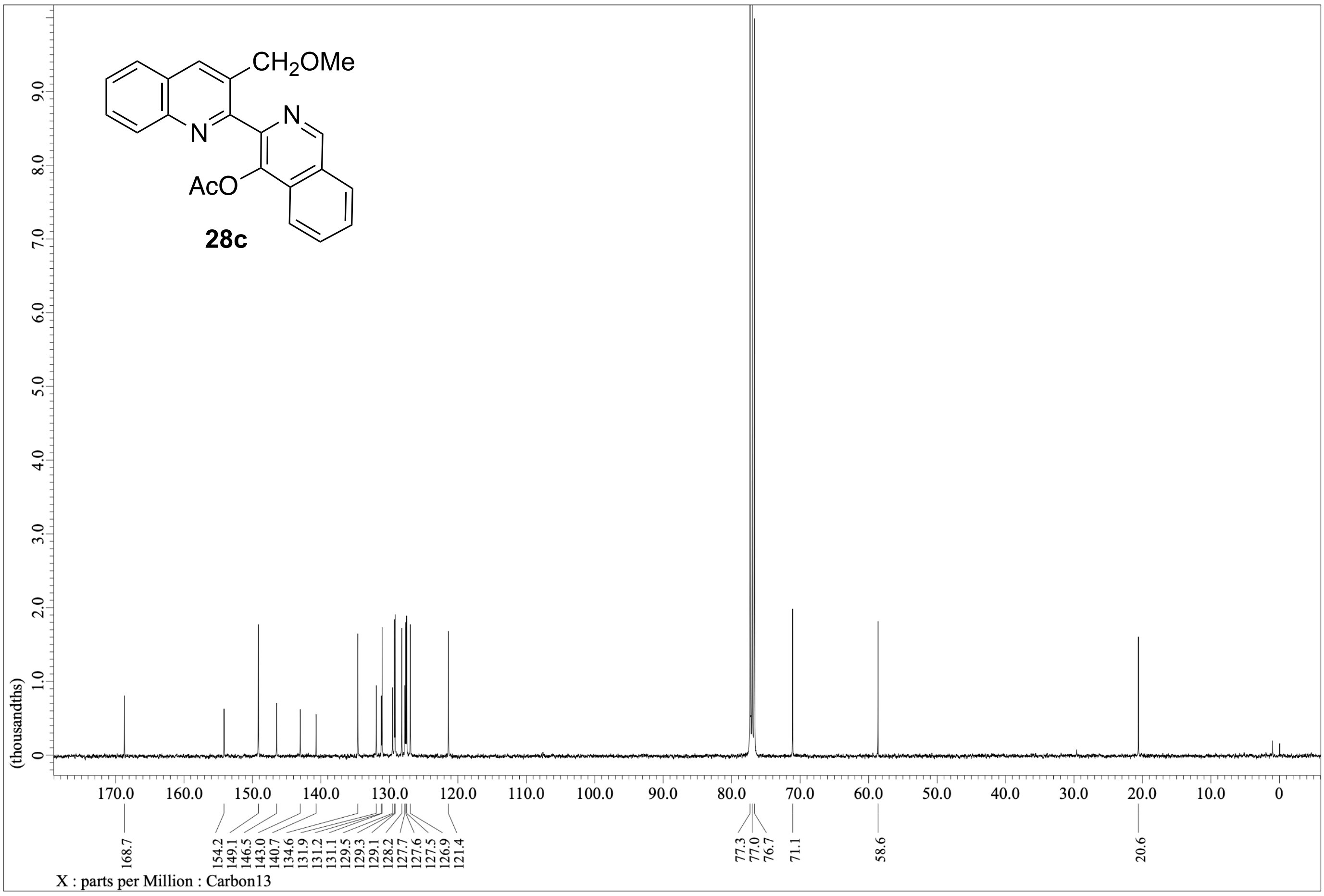

4-Hydroxy-3-(3-methoxymethylquinolin-2-yl)isoquinoline (29)

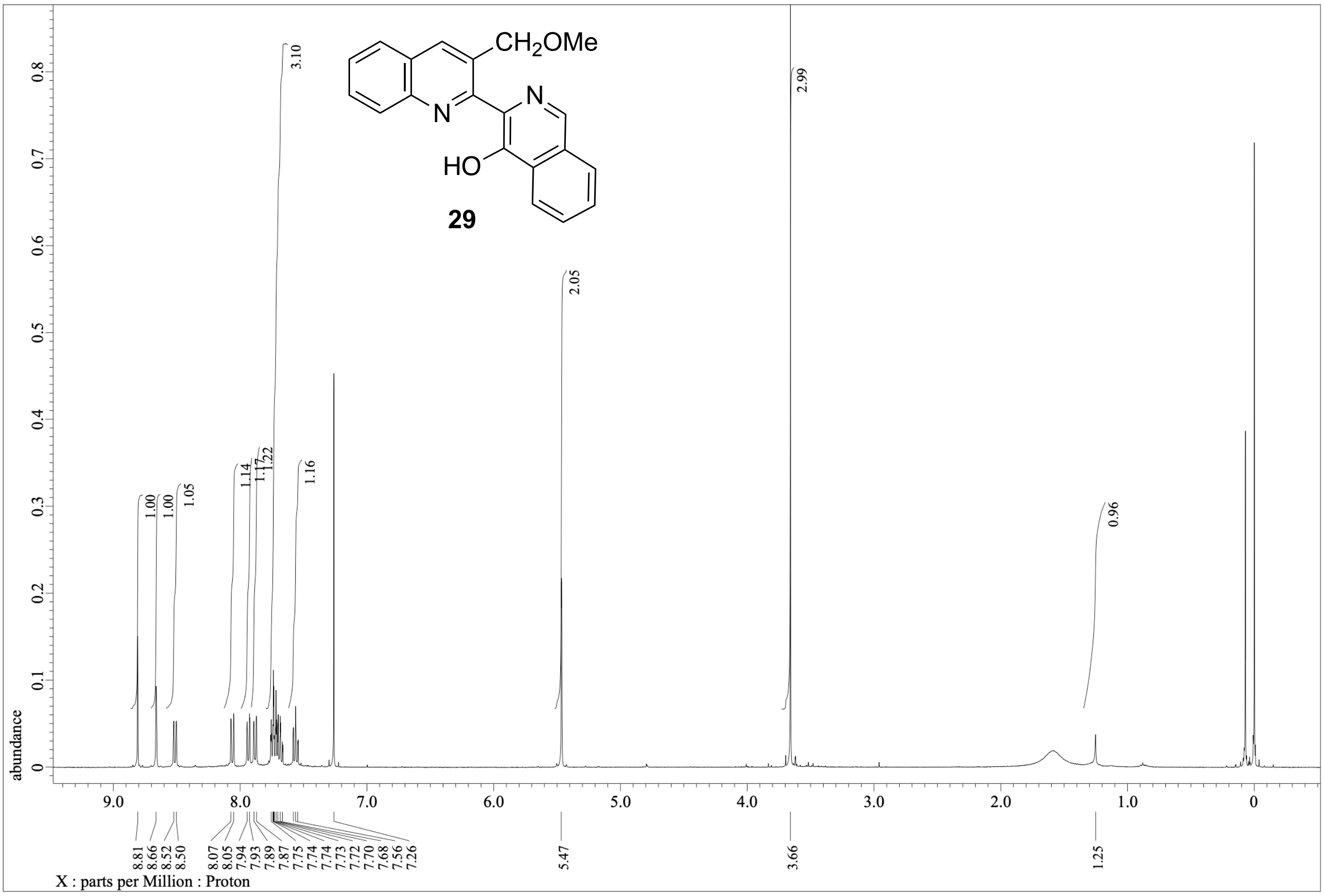

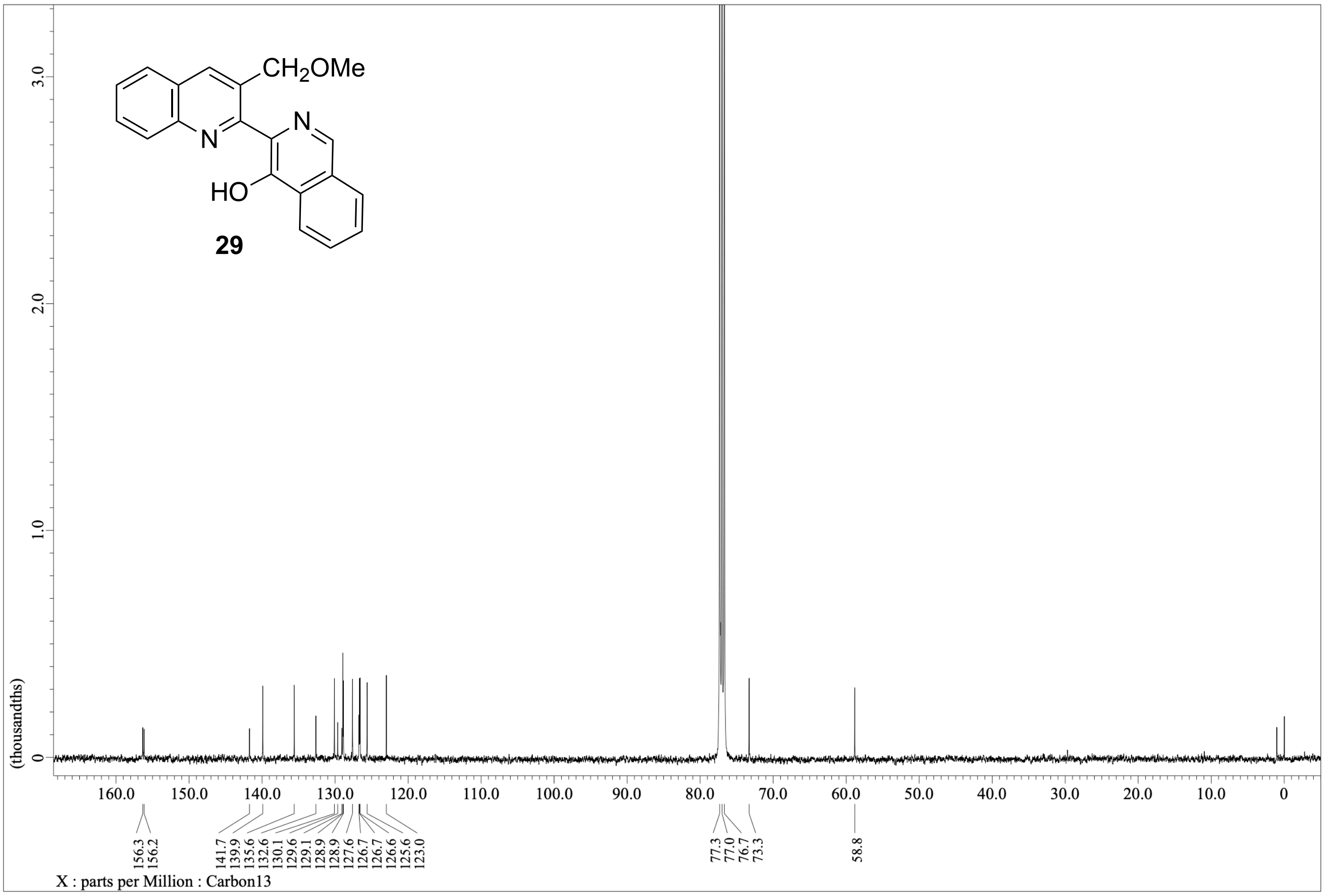

Rosettacin (5)

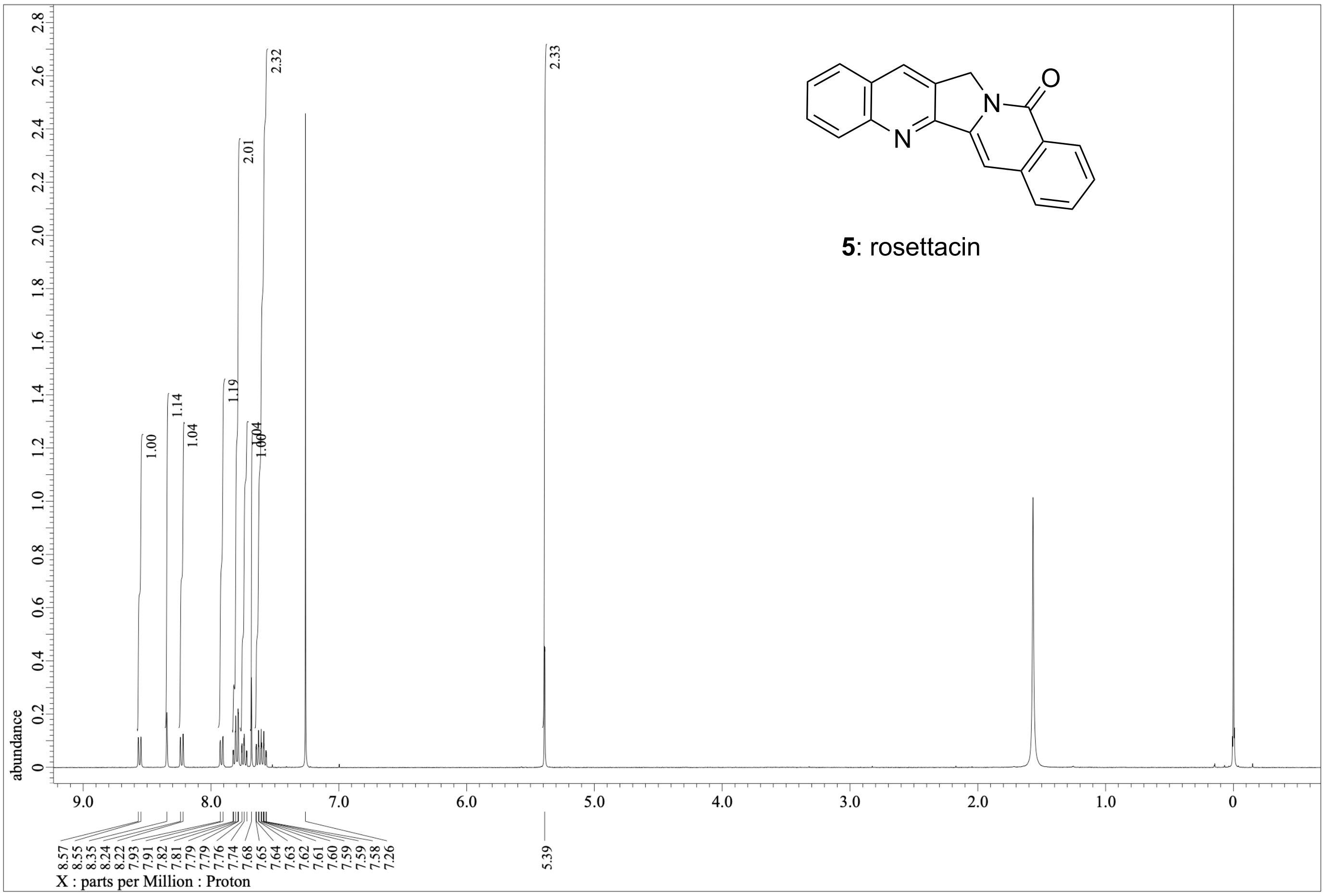

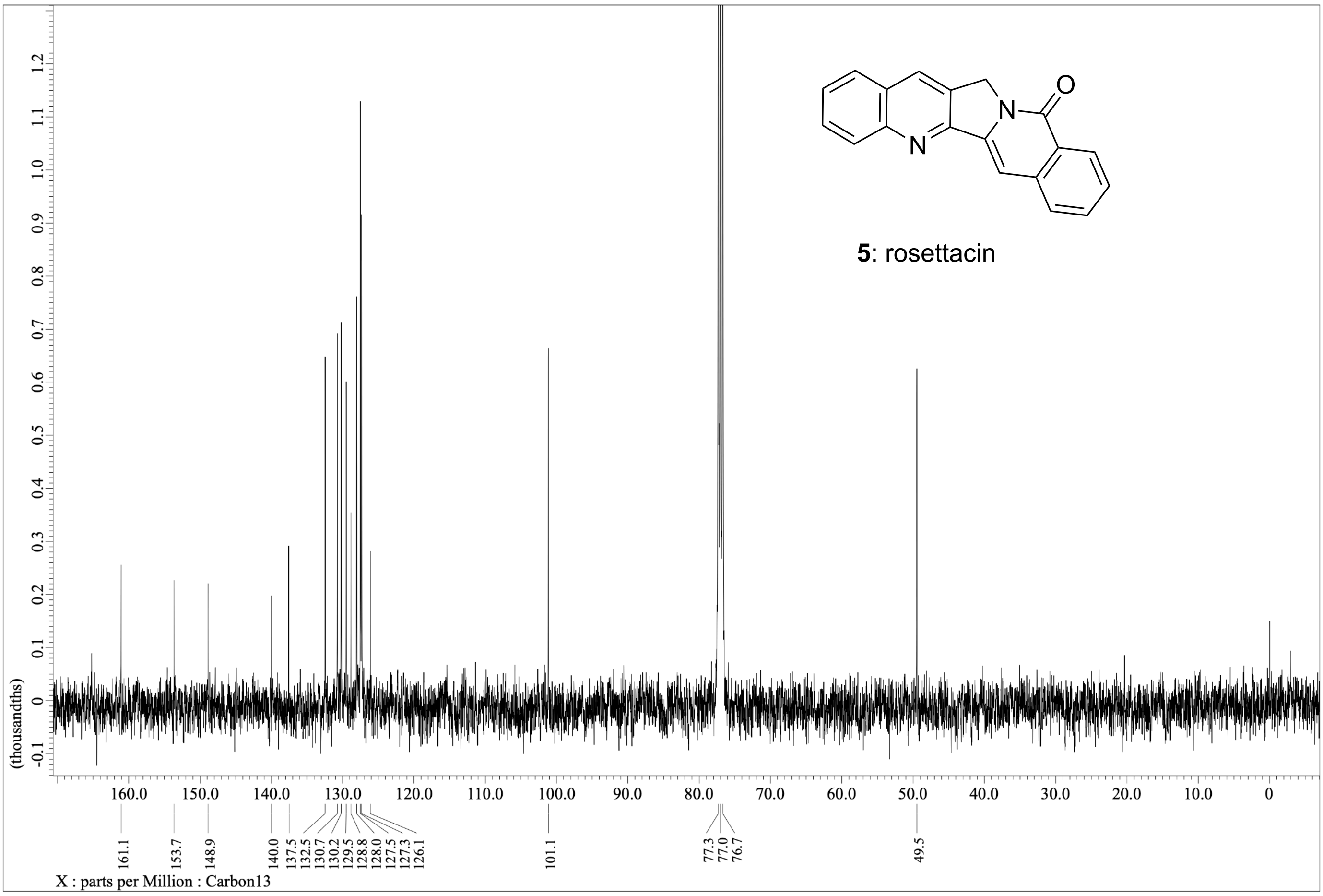

3-Methoxymethyloxymethyl-2-[(3-methoxymethylquinolin-2-yl)ethynyl]benzaldehyde (31)

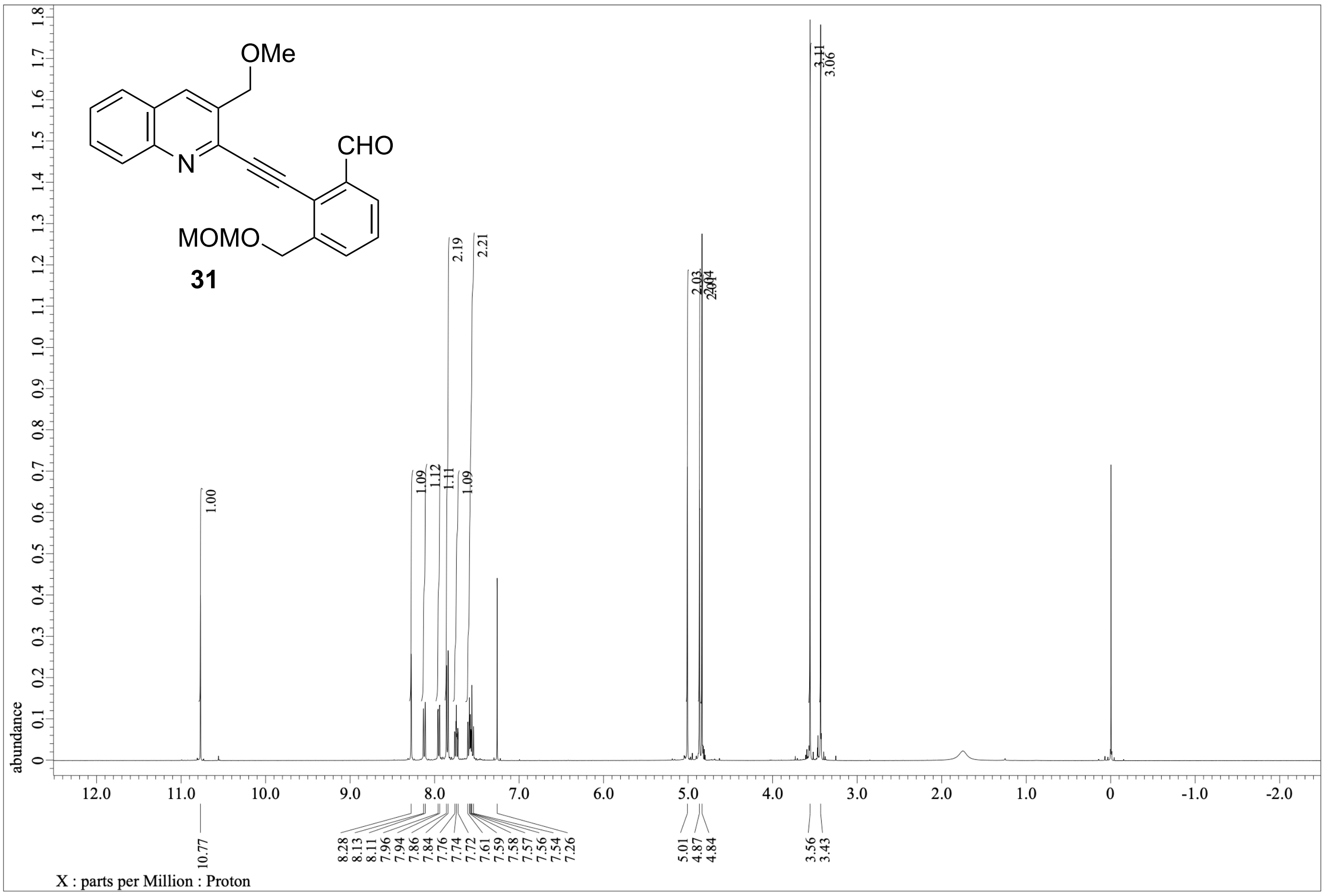

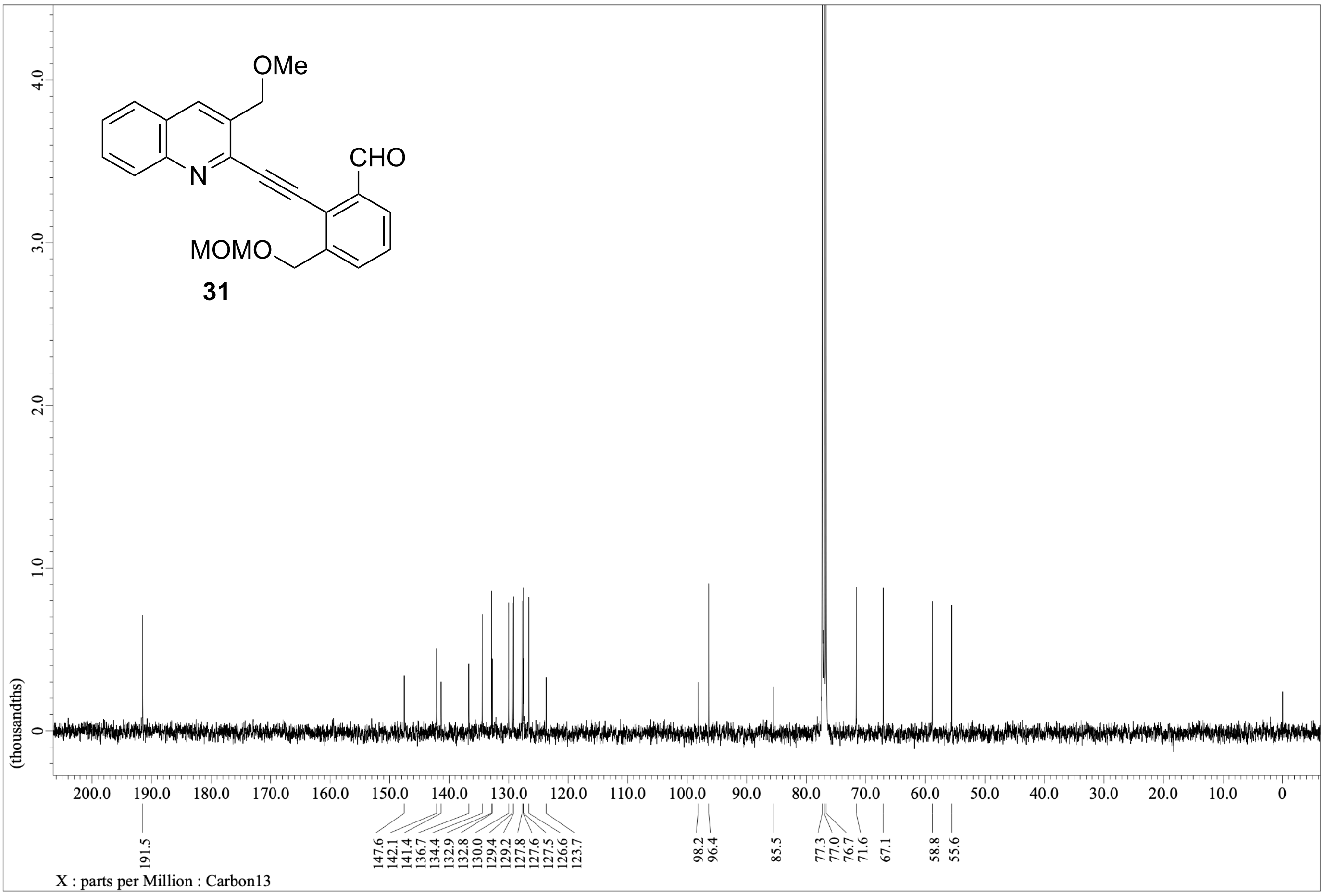

3-Methoxymethyloxymethyl-2-[(3-methoxymethylquinolin-2-yl)ethynyl]benzaldehyde oxime (32)

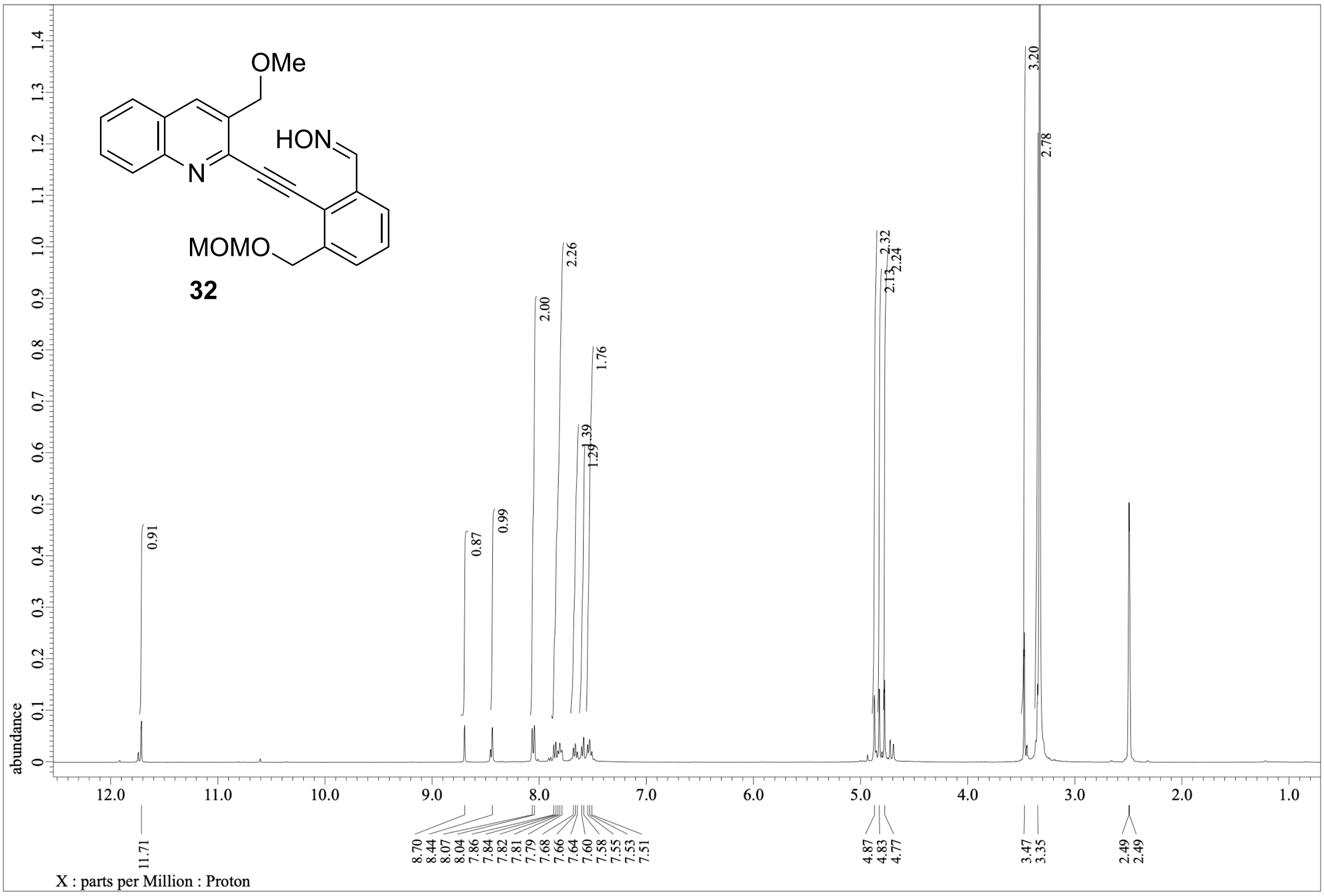

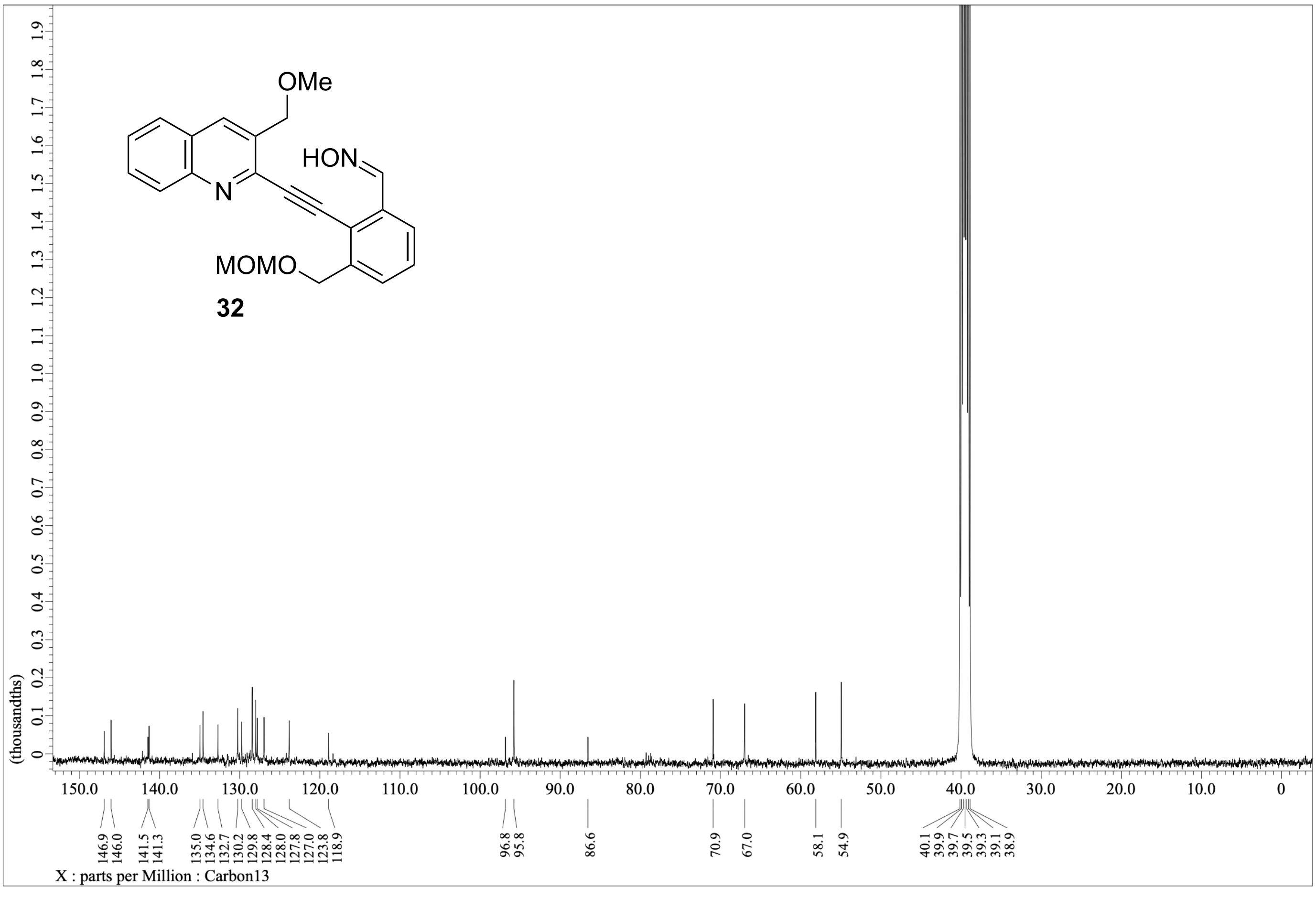

5-Methoxymethyloxymethyl-3-(3-methoxymethylquinolin-2-yl)isoquinolin-1-one (34)

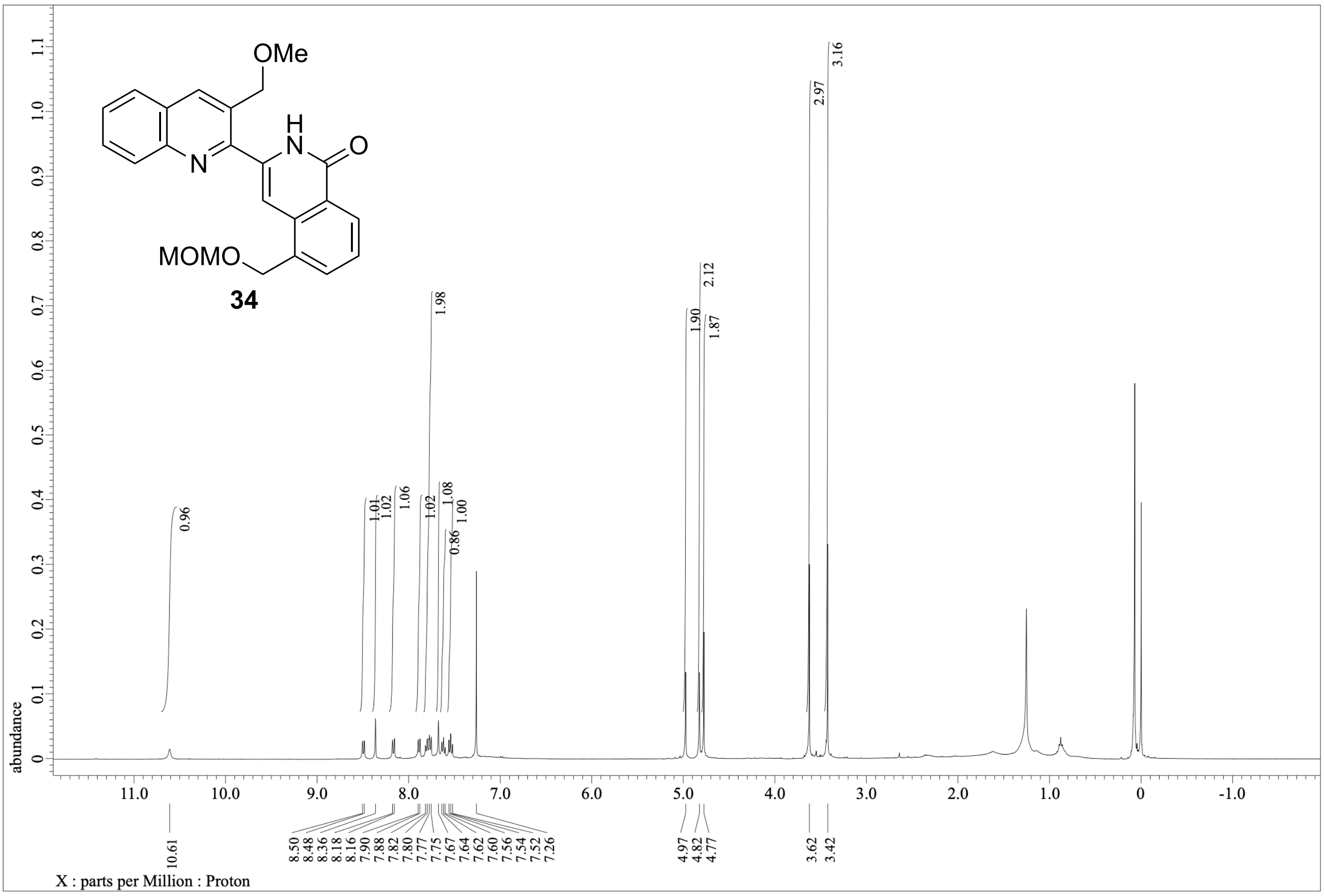

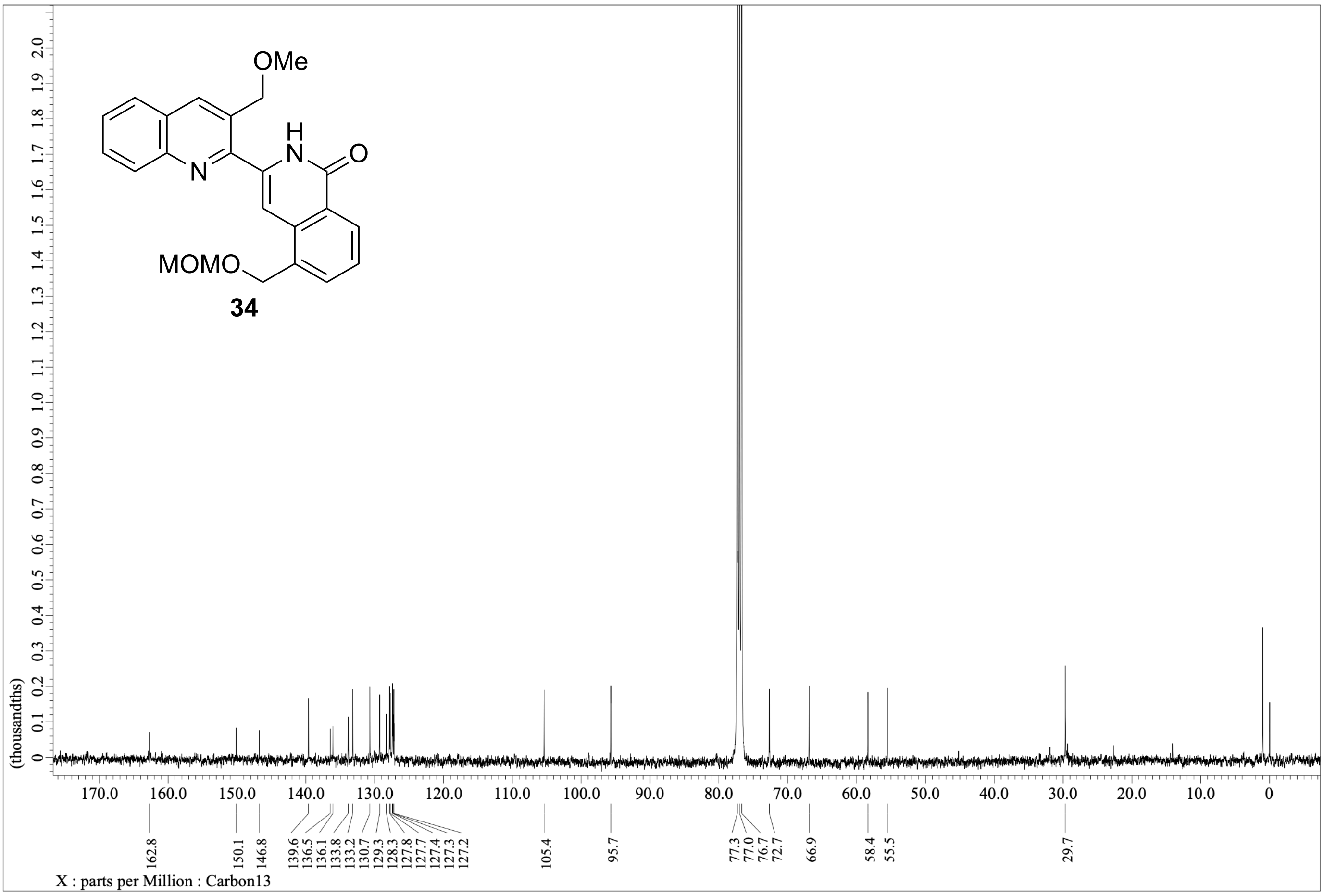

4-Acetoxy-5-methoxymethyloxymethyl-3-(3-methoxymethylquinolin-2-yl)isoquinoline (35)

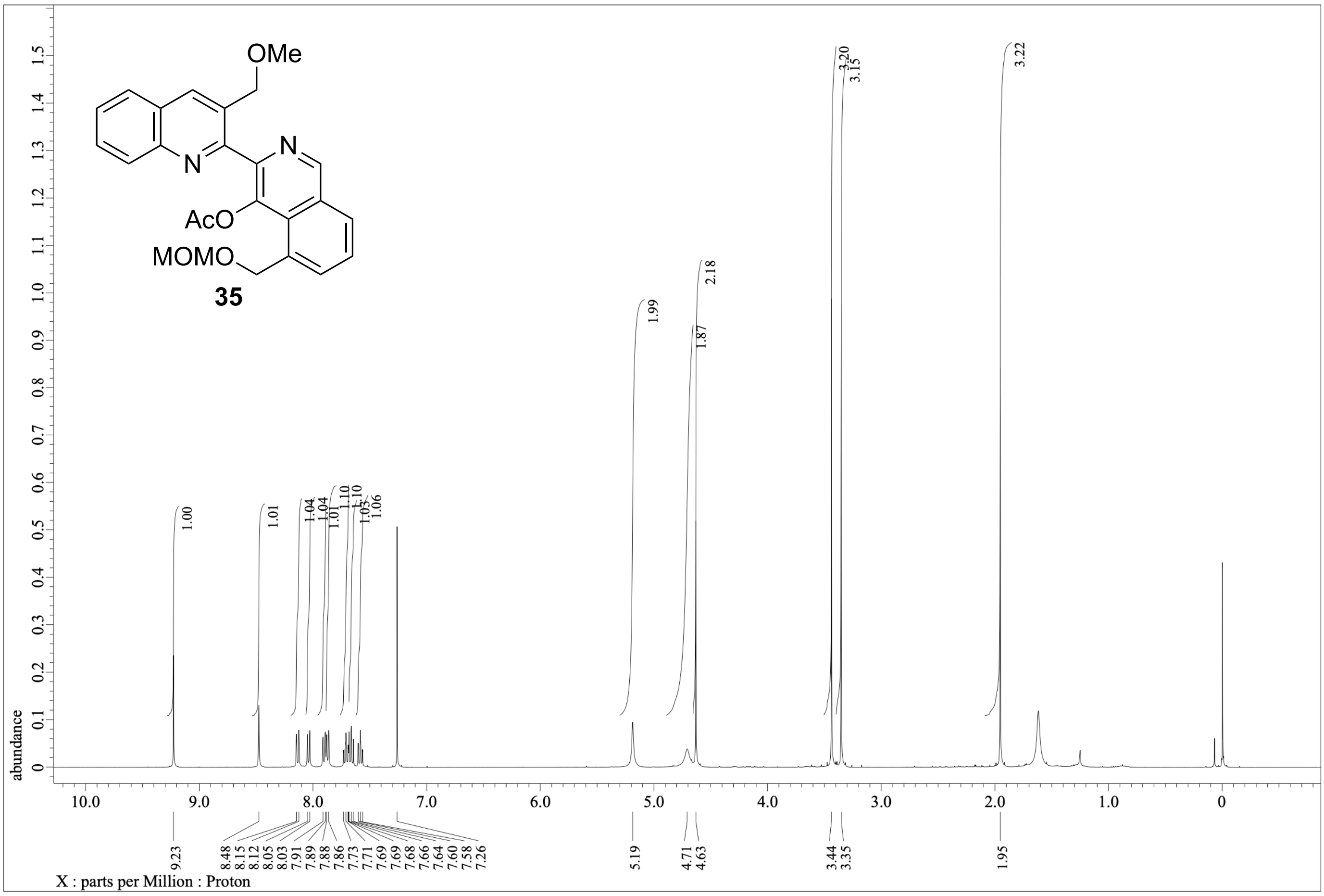

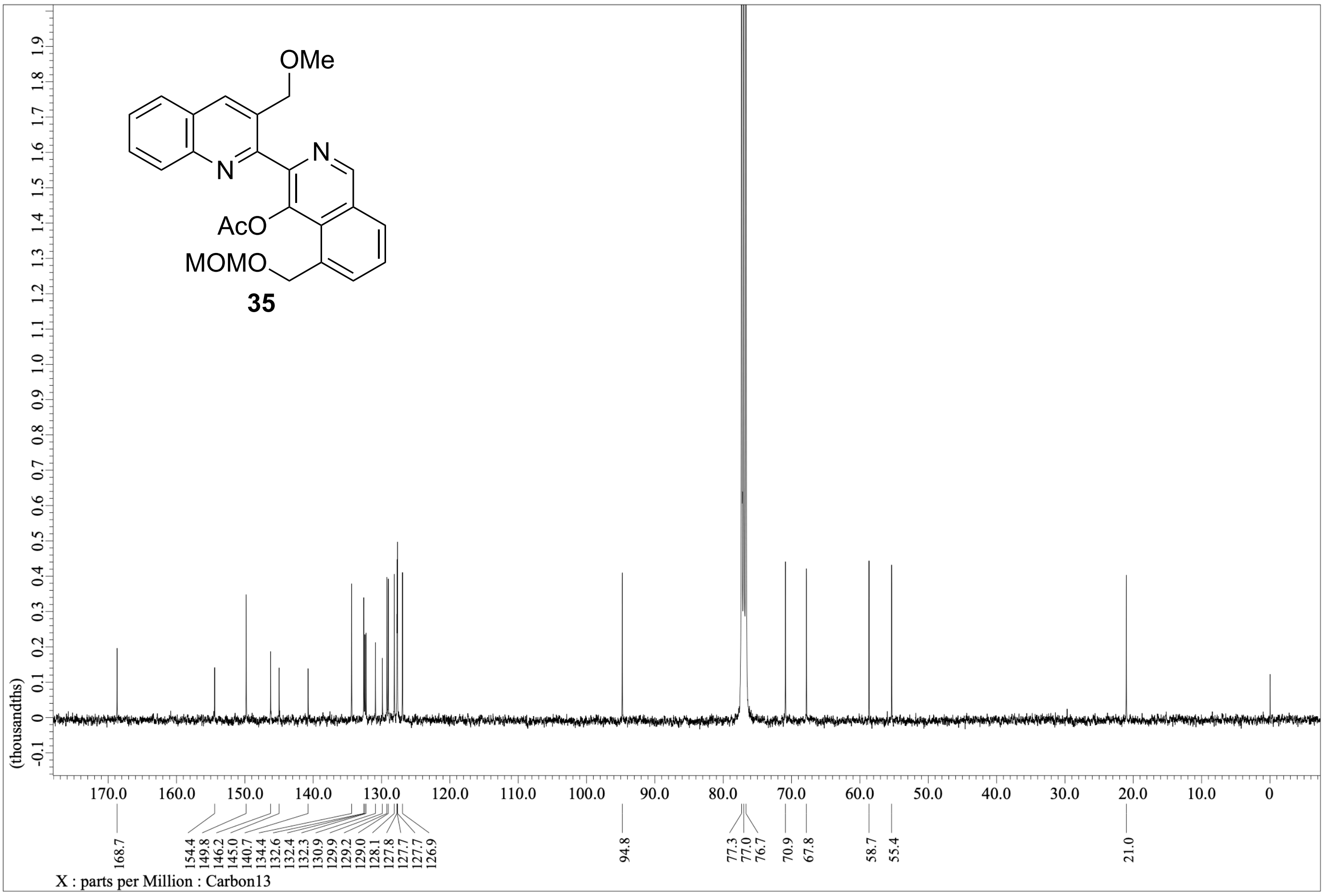

Acuminatine (7)

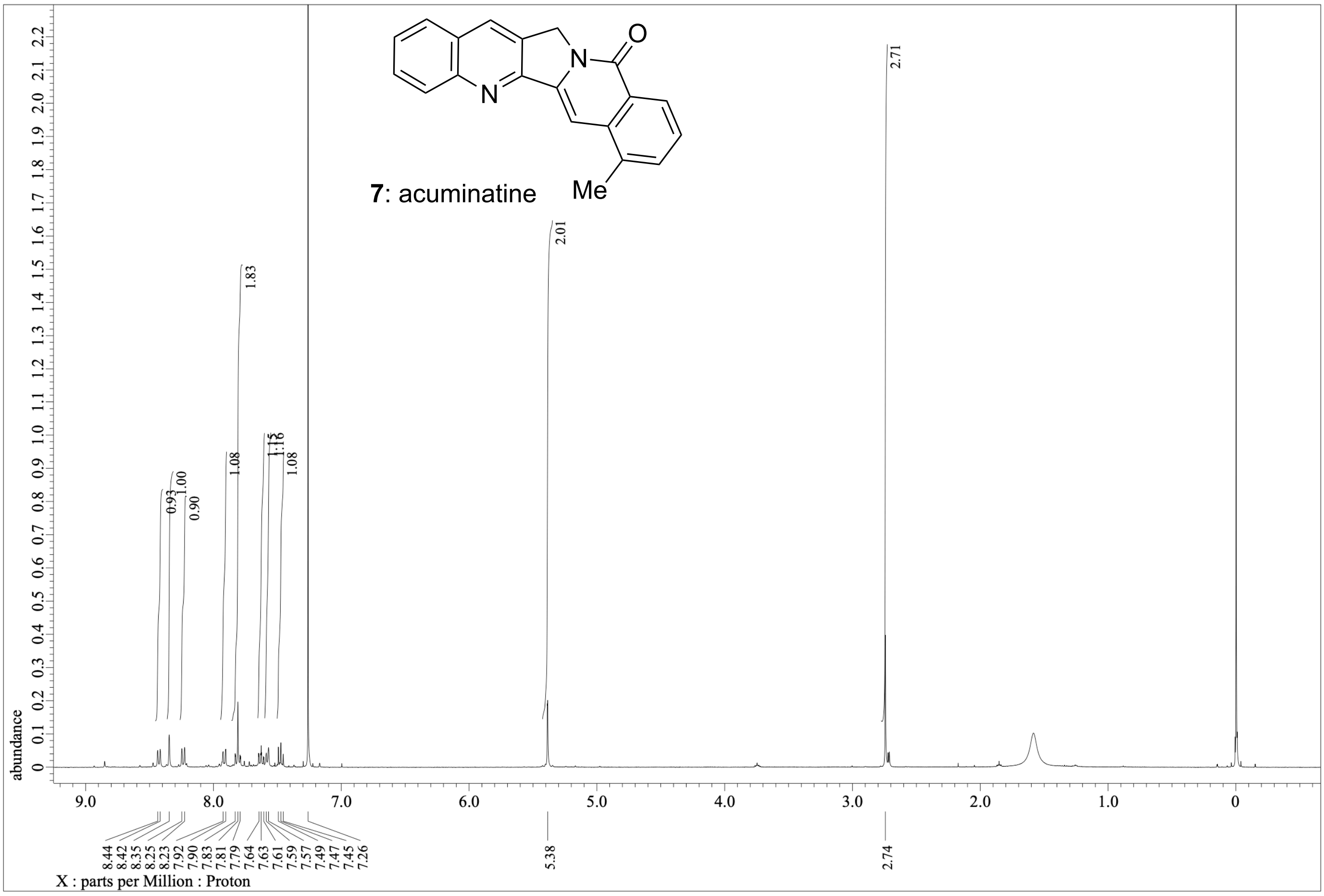

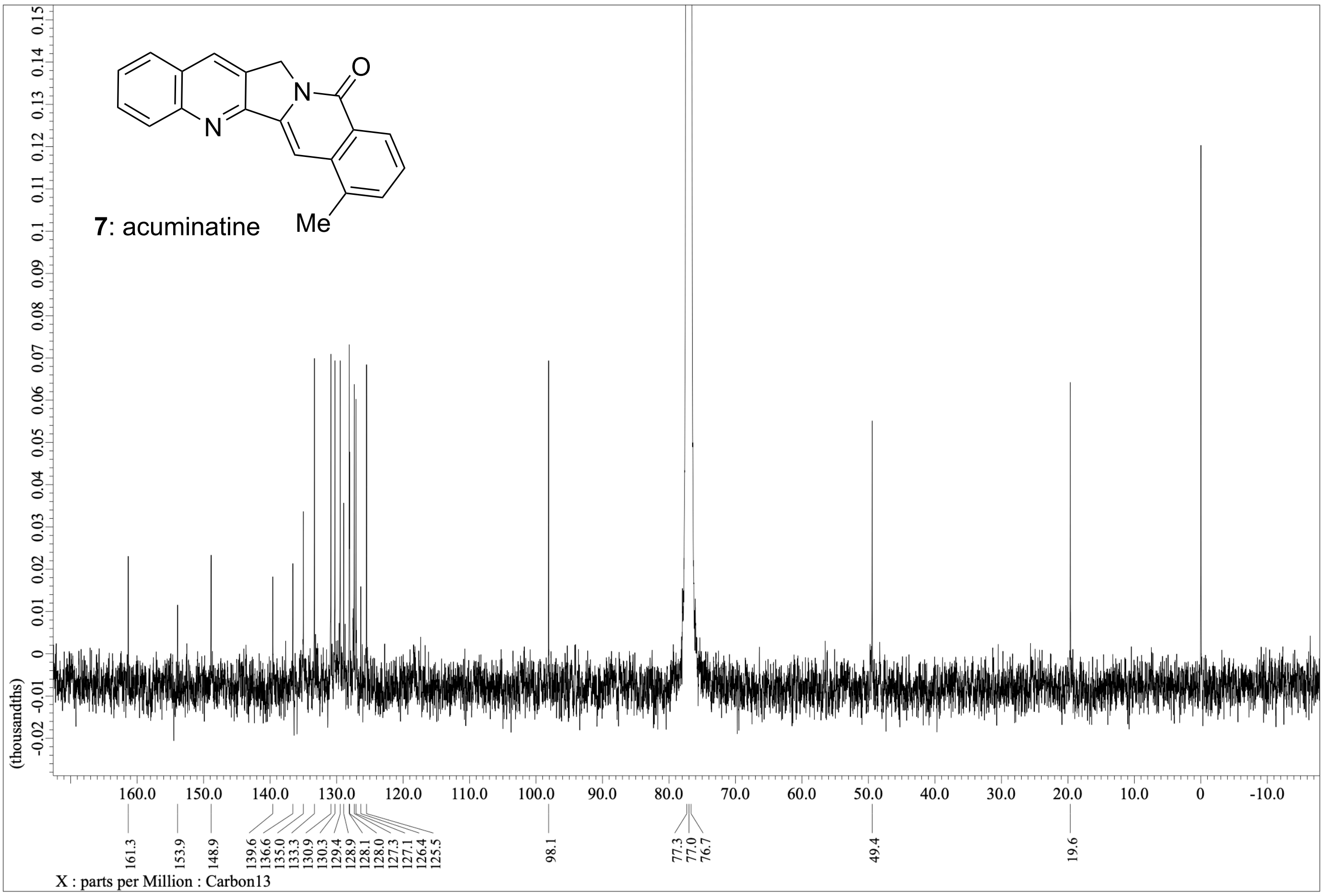

Supplement: Supplementary file 1 [file molecules-28-04059-s001.zip › molecules-2383974-supplementary.pdf]
